# Achieving high diet quality at eating occasions: findings from a nationally representative study of Australian adults

**DOI:** 10.1017/S0007114523002325

**Published:** 2024-03-14

**Authors:** Nancy R. Tran, Rebecca M. Leech, Katherine M. Livingstone, Sarah A. McNaughton

**Affiliations:** Institute for Physical Activity and Nutrition (IPAN), School of Exercise and Nutrition Sciences, Deakin University, Burwood, VIC, Australia

**Keywords:** Food consumption, Eating occasion, Eating patterns, Meals, 24-h recall, Diet quality, Snacks, Dietary guidelines

## Abstract

This study examined differences in food groups consumed at eating occasions by the level of adherence to dietary guidelines in Australian adults (≤19 years) and whether consumption differed with respect to age, sex and education levels. Secondary analysis of the 2011–2012 National Nutrition and Physical Activity Survey (*n* 9054) was performed, using one 24-h dietary recall with self-reported eating occasions. Dietary Guideline Index scores were used to assess adherence to the 2013 Australian Dietary Guidelines. Mean differences (95 % CI) in servings of the five food groups and discretionary foods at eating occasions were estimated for adults with higher and lower diet quality, stratified by sex, age group and education. Using survey-based *t*-tests, differences of at least half a serving with *P* values < 0·05 were considered meaningful. Compared with adults with lower diet quality, women and men aged 19–50 years with higher diet quality consumed more serves of vegetables at dinner (mean difference (95 % CI), women; 1·0; 95 % CI (0·7, 1·2); men: 0·9; 95 % CI (0·6, 1·3)) and fewer serves of discretionary foods at snacks (women: −0·7; 95 % CI (−0·9, −0·5); men: −1·0; 95 % CI (−1·4, −0·7). Other food groups, such as grains, dairy products and alternatives, meats and alternatives, were not significantly different between adults with lower and higher diet quality, across any eating occasions and age groups. Discretionary food intake at lunch, dinner and snacks was consistently greater among adults with lower diet quality, regardless of education level. Our findings identify dinner and snacks as opportunities to increase vegetable intake and reduce discretionary food intake, respectively.

Globally, a suboptimal diet increases risk factors for diet-related chronic diseases and is responsible for 22 % of all deaths and 15 % of all disability-adjusted life-years^(1)^. High BMI is one of the risk factors that are modifiable by improving lifestyle behaviour. In Australia, high BMI was the leading risk factor contributing to non-fatal burden (living with disease) and the second leading risk factor for fatal burden with 16 400 deaths (10 % of all deaths) in 2018^(2)^. Healthy eating messages and dietary guidelines form a key component of Australia’s prevention strategies to deal with growing public health challenges relating to high BMI.

The Australian Dietary Guidelines (ADG) recommend eating a wide variety of foods from five food groups (grain foods, vegetables (and legumes), fruits, dairy products and alternatives and meats and alternatives) to achieve a higher diet quality. Other healthy eating habits include selecting wholegrain and/or high-fibre grains, lean meats and reduced-fat dairy foods, drinking plenty of water and limiting discretionary foods/beverages that are high in energy, saturated fat, added sugars and/or salt or alcohol. However, the usual intake of five food groups for both men and women of all ages was well below the ADG recommended level in 2011–2012^(3)^. For example, 49 % did not eat the recommended two serves of fruit, and 92 % did not eat the recommended 5 to 6 serves of vegetables. Discretionary food intake also exceeded the maximum recommendation of 0 to 3 serves a day (where 600 kJ is equivalent to 1 serve); on average, adults consumed between 5 and 7 serves/d^(4)^.

Prior research on healthy dietary patterns has often focused on analysing overall daily intake, with few studies considering consumption patterns at eating occasions^(5)^. Identifying foods and beverages consumed at eating occasions is key to understanding food behaviour as it can assist with messages and translation to the public^(6)^. Eating occasion is a term used in research on eating patterns to describe the foods and beverages eaten together at meals and snacks^(7)^. The characteristics of each eating occasion during the day can provide a detailed picture of how their dietary behaviour differs, leading to more targeted intervention strategies through meal- and snack-based advice.

There are only a small number of studies that have examined variations in food consumption at eating occasions and even fewer studies involving Australian populations. A review of international literature on meal and snack consumption provides contrasting findings where snacking seems to provide valuable nutrients in healthy individuals while often contributes excessive energy with limited nutrition, especially in people living with obesity^(8)^. A recent study investigating Japanese adults’ dietary patterns found that distinctive meal-based dietary patterns at different eating occasions will lead to different diet quality^(9)^. In Australia, Rebuli *et al.* examined the average percentage of the ADG daily target for the five-food groups consumed at each eating occasion^(10)^. Breakfast contributed an average of 18·6–47·0 % towards the daily grain targets, 11·2–29·3 % for fruit and 10·2–35·3 % for dairy product and alternative foods. At lunches, Australian adults consumed an average of 17·9–38·2 % towards the recommended daily grains intake and only 12·4–19·4 % towards vegetable intake. Dinners contributed 17·4–39·4 % for grains food groups, and vegetable consumption was only 18·5–40·8 %, meats and alternatives at 30·7–56·5 % and fruits at 4·3–29·5 %, respectively. Another study also showed that meats and alternatives food groups were mainly consumed at lunch and dinner, with males being more likely to consume red meat, poultry and processed meat than females at lunch^(11)^. Furthermore, fish and seafood consumption was associated with the least disadvantaged socioeconomic position^(11)^.

Existing studies have demonstrated that Australians consume well above the amount recommended for discretionary foods. Fayet-Moore *et al.* showed that the population average intake of discretionary food was five serves/d, with 45 % and 30 % of that intake consumed at lunch and dinner (combined) and snacks, respectively^(12)^. Using the same national population survey data, the findings from Rebuli *et al.* were consistent with Fayet-Moore *et al.* where discretionary foods were consumed at all eating occasions (breakfast, lunch, dinner and snacks) and made up a large contribution to total energy intake^(10)^, irrespective of sex and age group.

While a small number of previous studies have examined food intake at eating occasions^(10–12)^, little is known about the differences in food intake at eating occasions between those with higher diet quality (i.e., higher adherence to the ADG recommendations) and those with lower diet quality. The difference in food intake at each occasion between adults with higher *v*. lower diet quality can describe eating patterns, potentially offering insights to encourage more Australians to follow the ADG. Therefore, the primary aim of this study is to examine differences in foods and beverages consumed at eating occasions by the level of adherence to dietary guidelines in a nationally representative sample of Australian adults (19 years and over), with a secondary aim to examine whether consumption differed with respect to age, sex and education levels.

## Methods

### Participants and procedures

The National Nutrition and Physical Activity Survey (2011–2012) is the latest nationally representative population nutritional survey in Australia. This data is a component of the 2011–2012 Australian Health Survey, collected by the Australian Bureau of Statistics (ABS)^(13)^. Details of the 2011–2012 the National Nutrition and Physical Activity Survey have been published elsewhere^(13)^. Briefly, the National Nutrition and Physical Activity Survey is a nationally representative, cross-sectional survey that measures foods, beverages and supplements intake, as well as general information on the Australian population^(13)^. The ABS selected the sample population using a stratified multistage area sample of private dwellings with a response rate of 77 %. Individuals who lived in non-private dwellings such as boarding schools, prisons, hospitals and nursing homes were excluded from the survey. Within each household, one adult and one child were selected for the survey. Household and person weights were calculated to ensure appropriate representations of the total population. A total of 12 336 households were approached for inclusion, with a total of 12 153 participants included.

**Fig. 1.**
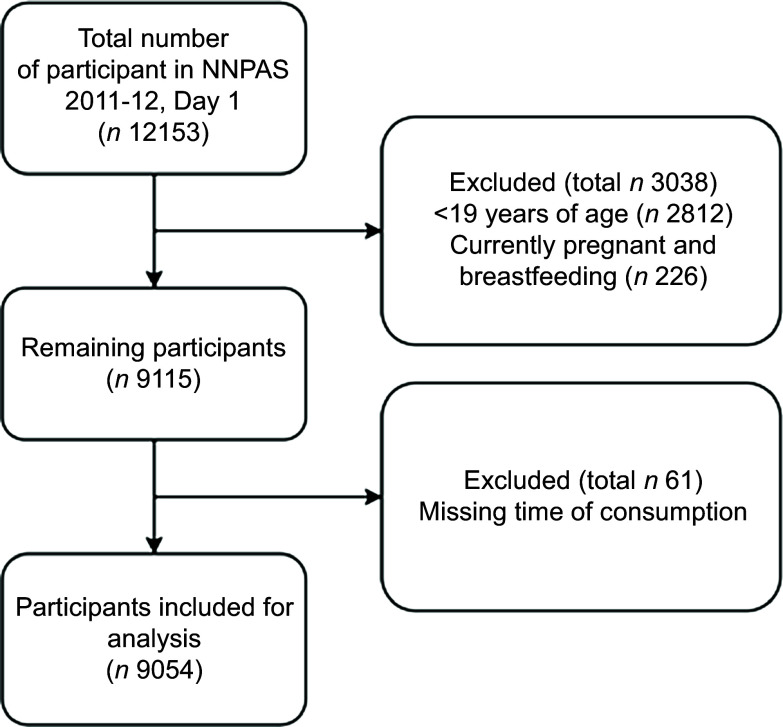
Flow diagram of participants included in the analysis.

For this analysis, respondents were excluded if they: (i) were 18 years of age or younger; (ii) were pregnant and/or breast-feeding or (iii) had missing data for time of consumption (Fig. 1). The data from the remaining respondents were used in this study. An exemption from ethics review was approved by the Deakin University Human Research Ethics Committee for this analysis of pre-existing and non-identifiable data (DUHREC); application 2018–415).

## Measures

### Dietary intake assessment and classification of food groups

Dietary intake was collected via two 24-h dietary recalls^(14)^. The first recall collected all foods and beverages consumed on the day before the interview. The second recall was performed via telephone (computer-assisted telephone interview) and, when possible, at least 8 d after the first recall interview. This analysis used the first day of dietary recall to maintain the national representativeness of the sample across age groups, as only a subset of the population completed the second dietary recall (63·6 %). The dietary recall method was based on the National Health and Nutrition Examination Survey 5-step Automated Multiple-Pass Method. Information used for this analysis are participant-identified labels of eating occasion, time of consumption, food descriptions and amount of food eaten.

The Australian Food, Supplement and Nutrient Database 2011–2013 (AUSNUT13) was used to determine the amount of food consumed. AUSNUT13 is a database developed by the Food Standards Australia and New Zealand to analyse the 24-h recall data^(15)^.

Foods and beverages reported by respondents were grouped by the ABS according to five food groups recommended by the ADG and a discretionary food group. The ADG Food Group database was used to estimate food intakes of the five food groups^(16)^, while discretionary foods are categorised using ABS-provided classification^(17)^. The food groups are (i) vegetables: including different types and colours of vegetables and legumes/beans; (ii) fruits: including different types of fruits, juices and dried fruits, such as varieties of pome fruits, citrus fruits, stone fruit, tropical fruit, berries and other fruits; (iii) grains: mostly wholegrain and/or high cereal fibre varieties; (iv) meats and alternatives: lean meats and poultry, fish, eggs, tofu, nuts and seeds and legumes/beans; (v) dairy products and alternatives: milk, yoghurt, cheese and/or their alternatives, mostly reduced fat and (vi) discretionary (energy dense, nutrient poor): foods that contain a high level of saturated fat, added salt, added sugar and alcohol such as potato chips, cakes, processed meats and sugar-sweetened beverages. The amount of intake was calculated as number of serves consumed per eating occasion, according to the ABS classification^(14)^.

### Diet quality

The Dietary Guideline Index (DGI) was used as a measure of diet quality and to assess adherence to the food-based recommendation in the 2013 ADG^(18–20)^. The DGI has been adapted for application in 24-h recall data and comprises 23 items (Additional file 1). The 23 items capture important food choices that reflect adherence to the 2013 ADG recommendations. Each item has cut-offs used to obtain the maximum score (10 points); they were guided by the age and sex-specific food-based daily recommendations outlined in the ADG. The DGI scores ranged from a minimum of 0 to a maximum of 130, with a higher score implying better diet quality^(19)^. Respondents were stratified according to the DGI score of: (i) higher level of adherence or higher diet quality: those with the top tertile of the DGI score; and (ii) lower level of adherence or lower diet quality: all other scores that were not in the top tertile.

### Categorisation of eating occasions

Respondents self-reported the name of eating occasion and eating time when each food item was consumed. Reported eating occasions were chosen from a list of pre-defined terms provided by the ABS: breakfast, morning tea, lunch, dinner, supper, afternoon tea, snacks, drink/beverage, extended consumption (i.e. eating occasions that extend over time) and other/I do not know. For this analysis, eating occasions were classified as breakfast, lunch, dinner or snacks, based on previously published approaches^(7)^. Breakfast included all foods and drinks items that were reported as breakfast and/or brunch. Lunch included items that were reported as lunch. Dinner included items that were reported as dinner and/or supper. Snacks included items that were reported as snacks, morning tea or afternoon tea^(7)^. Items that were identified as extended consumption, other, I do not know/not determined were considered as either breakfast, lunch, dinner or snacks when: (i) intake occurred at the same time as breakfast, lunch, dinner or snacks or (ii) intake occurred ≤ 15 min after breakfast, lunch, dinner or snacks, respectively, because we inferred that these intakes are a continuation of the preceding eating occasion^(7)^. Food items that occurred after dinner were categorised as snacks if the participants defined that eating occasion as a snack. And, for this analysis, the time of day (timing) of eating occasion was not examined.

### Socio-demographic characteristics

Socio-demographic data were collected by ABS-trained and experienced interviewers. Information collected included sex, age, country of birth, marital status, number of persons in the household, geographical region of residence, household income, labour force status, duration of unemployment, shift work, level of education, area-level disadvantage and food security. For this analysis, age was categorised as 19–51, 51–70 and 71 years and over in alignment with age categories used in Australian nutrition recommendations^(21)^. Country of birth was categorised by the ABS as Australia, main English-speaking countries or other. Marital status was categorised as married or not married. The number of persons in the household ranged from 1 to 6 or more. The geographical region of residence was assessed using the Australian Statistical Geography Standard Remoteness areas categories (2011) and was divided into three categories – major cities of Australia, inner regional Australia and others. Household income was defined as the gross weekly combined equivalised income of all household members and was divided into quintiles: lowest 20 % to highest 20 %. Labour force status was defined as employed, unemployed and not in the labour force. Duration of unemployment was categorised as under 4 weeks, 4 weeks or more and not applicable. Shift work was categorised as yes, no or not applicable. The level of education was categorised as low (incomplete high school or less), medium (complete high school or incomplete high school and/or certificate/diploma) and high (tertiary qualification). Area-level disadvantage was assessed by the socio-economic indexes for areas provided in the survey. Area-level disadvantage was divided into quintiles ranging from most disadvantaged (lowest 20 %) to least disadvantaged (highest 20 %). Food security was defined by respondents’ answers to the question ‘Whether ran out of food in the last 12 months and could not afford to buy more’, and answers were categorised as yes or no.

### Statistical analysis

Descriptive statistics were used to provide summary estimates of the sample characteristics, with sample stratified by diet quality, as described above. Differences in sample characteristics between lower and higher diet quality were assessed using survey design adjusted *F*-test and *χ* ^2^ test as appropriate. The weighted proportions of consumers and non-consumers were calculated to examine the degree of non-consumption of food groups at breakfast, lunch, dinner and snacks. Eating occasions with no food group consumption were excluded. The mean serves (95 % CI) for food groups were stratified by diet quality and reported separately for men and women and across age groups. Survey *F*-tests were used to estimate mean differences in serves of food group consumption between adults with higher and lower diet quality. Linear regression models, adjusted for age group, were used to estimate the marginal means (95% CI) of food group consumption among adults with higher *v*. lower diet quality, stratified by education level^(22)^. Bonferroni correction was used to adjust the *P* values when comparing marginal means. Differences of ≥0·5 serve with a *P* value < 0·05 were considered meaningful differences. All results presented were weighted using the person-specific survey weights and replicate weights (Jackknife delete-1 method) provided by the ABS, to account for selection probability and the effect of complex sampling procedures. Statistical analysis was performed using RStudio (R 4.1.2) and Stata 17 software.

## Results

### Sample characteristics

Of 9054 adults included in the analysis, 66·8 % were classified as having lower level of adherence to the ADG (in other words, lower diet quality). Compared with populations with lower diet quality (as shown in Table 1), those demonstrating higher diet quality were more likely to be female (*P* < 0·001), married (*P* = 0·006), have higher educational attainment (*P* < 0·001), have higher household income (*P* = 0·003), reported lower levels of food insecurity (*P* < 0·001) and had lower socio-economic disadvantage as indicated by their socio-economic indexes for areas scores (*P* < 0·001). No significant differences were found for other characteristics.

**Table 1.** Characteristics of 9054 adults aged 19+ by diet quality*, using dietary guideline index score

*n* (person)	Lower diet quality		Higher diet quality		*P* value†
	*n* 6037	% 66·8	*n* 3017	% 33·2	
Age					0·13
19–50	3240	58·9	1652	60·3	
51–70	1984	30·3	906	28·0	
71+	813	10·8	459	11·7	
Sex					<0·001
Male	3018	53·4	1227	44·9	
Female	3019	46·6	1790	55·1	
Country of birth					0·023
Australia	4347	69·8	2063	66·5	
Other	1690	30·2	954	33·5	
Education level					<0·001
Low	1868	28·4	705	20·5	
Medium	2848	49·7	1342	48·0	
High	1321	21·9	970	31·5	
Number of persons in household					0·94
1 person	1672	14·3	838	14·4	
2 persons	2138	34·8	1054	35·8	
3 persons	898	18·4	434	18·3	
4 persons	875	21·7	466	21·2	
5 persons	336	8·0	160	7·3	
6 or more persons	118	2·8	65	3·0	
Household income					0·003
First quintile	1224	17·2	511	14·5	
Second quintile	1002	15·6	482	15·1	
Third quintile	978	17·3	490	17·3	
Fourth quintile	1148	18·5	604	19·8	
Fifth quintile	1064	17·3	650	21·1	
Not known	621	14·2	280	12·2	
Labour force status					0·041
Employed	3825	66·2	1941	68·7	
Unemployed	153	3·0	53	1·8	
Not in the labour force	2059	30·8	1023	29·6	
Duration of unemployment					0·013
Under 4 weeks	33	0·9	8	0·2	
4 weeks or more	120	2·2	45	1·6	
Not applicable	5884	97·0	2964	98·2	
Shift work‡					0·24
Yes	606	11·0	288	10·8	
No	3219	55·2	1653	57·9	
Not applicable	2212	33·8	1076	31·3	
Food security					<0·001
Yes	314	4·2	82	2·2	
No	5723	95·8	2935	97·8	
Social marital status					0·006
Married	3105	57·9	1657	61·8	
Not married	2932	42·1	1360	38·2	
Geographical region of residence					0·11
Major cities of Australia	3789	70·5	2030	73·3	
Inner regional Australia	1205	19·8	552	18·2	
Other	1043	9·7	435	8·6	
SEIFA§					<0·001
Lowest 20 %	1216	19·6	503	15·4	
Second quintile	1311	21·3	561	17·5	
Third quintile	1214	20·8	576	20·1	
Fourth quintile	999	17·6	596	21·3	
Highest 20 %	1297	20·7	781	25·7	
Eating occasions					<0·001
Breakfast	5210	23·5	2888	24·9	
Lunch	5144	23·9	2828	24·4	
Dinner	5698	25·9	2958	25·4	
Snack	5862	26·6	2956	25·3	
	Median	Q1–Q3	Median	Q1–Q3	
Dietary guideline index score	66·0	66·0–66·3	89·6	89·3–89·9	<0·001

* Higher diet quality – the top tertile of dietary guidelines index score (0–130) which assessed adherence to the Australian Dietary Guidelines. Lower diet quality – bottom two tertiles of the score.

† Differences between lower and higher level of adherence assessed using *t*-test for continuous variables with normal distribution, Kruskal–Wallis H test for continuous variable with non-normal distribution and Pearson’s *χ* ^2^ test for categorical variables.

‡ Did shift work in the last 4 weeks.

§ Socio-economic indexes for areas were divided into quintiles ranging from most disadvantaged (lowest 20 %) to least disadvantaged (highest 20 %).

### Consumers and non-consumers at eating occasions

**Fig. 2.**
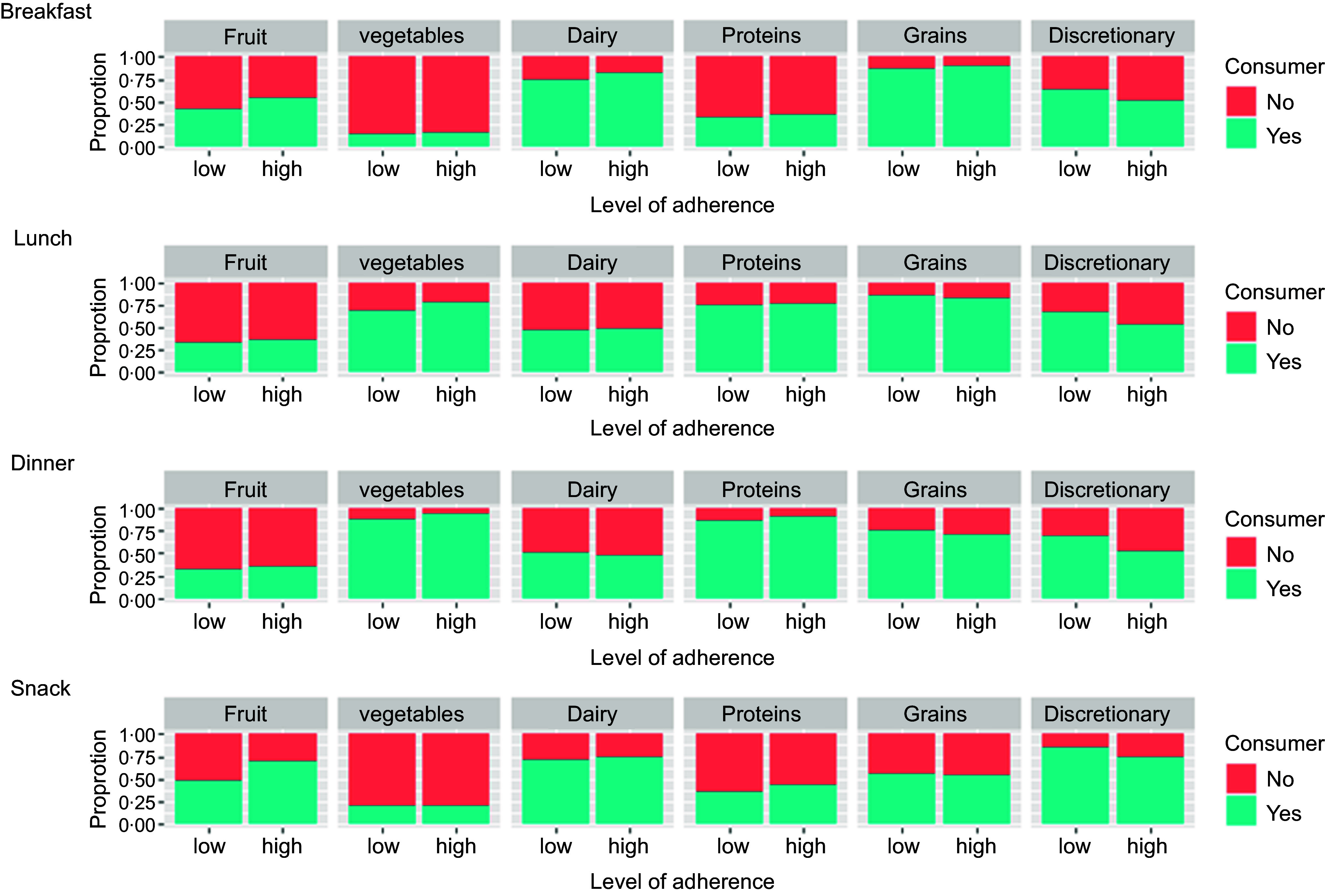
Weighted proportion of consumers of the five food group foods and discretionary foods at each eating occasion among adults with higher and lower levels of diet quality, as indicated by the dietary guidelines index score (*n* 9054).

Proportions of consumers and non-consumers of food groups at eating occasions were examined (Fig. 2 and Additional file 2). Regardless of diet quality, the proportions of adults who consumed vegetables and meat and alternatives foods were low at breakfast and snacks. A similar trend was observed for fruit at lunch and dinner, where only a small proportion of adults consumed fruit at all. Differences in the proportions of discretionary food consumption between lower and higher diet quality were observed across all eating occasions. Notably, populations with lower diet quality had a higher percentage of consumers of discretionary foods compared with non-consumers, while the opposite trend was observed for fruit consumption (higher proportion of consumers for higher diet quality) during breakfast and snacks.

### Differences in consumption stratified by age group

Table 2 presents the weighted mean difference (95 % CI) in serves of food group intakes among women with lower and higher diet quality (for weighted mean serves, refer to Additional file 3). Compared with women with lower diet quality, women aged between 19 and 50 years with higher diet quality consumed less discretionary foods at lunch, dinner and snacks (mean differences (MD): −0·7, −1·0 and −1·0 serves, respectively). They also consumed more vegetables at breakfast and dinner (MD: 0·6 and 1·0 serves). For women aged between 51 and 70 years, those with higher diet quality also consumed less discretionary foods at all eating occasions, especially for snacks (MD for breakfast, lunch, dinner, snacks: −0·2, −0·6, −0·7 and −1·1 serves). They also consumed more fruit at lunch (MD: 0·4 serves), more vegetables at lunch and dinner (MD: 0·7 and 1·0 serves) and more meat and alternatives foods for snacks (MD: 0·4 serves) than those with lower diet quality. Women above 70 years old with higher diet quality consumed less discretionary foods for lunch and snacks (MD: −0·4 and −0·6 serves) and more fruits and vegetables for dinner (MD: 0·4 and 0·8, respectively).

**Table 2.** Weighted mean differences in serves of food group consumption at eating occasions between Australian women with lower and higher level of diet quality*, stratified by age group (*n* 4809)

*n* (person)	19–50 years			51–70 years			71 years and over		
	2512			1556			741		
	Diff	95 % CI	*P*	Diff	95 % CI	*P*	Diff	95 % CI	*P*
Breakfast (serve/s)									
Fruits	0·1	−0·1, 0·2	0·471	0·2	0·0, 0·3	0·047	0·3	0·1, 0·6	0·010
Vegetables	**0·6**	**0·1, 1·0**	**0·010**	0·2	−0·4, 0·7	0·550	0·5	−0·3, 1·3	0·197
Dairy products	0·1	0·0, 0·2	0·051	0·1	0·0, 0·2	0·004	0·1	0·0, 0·2	0·039
Proteins	0·0	−0·1, 0·1	0·953	0·0	−0·1, 0·2	0·562	0·1	−0·1, 0·3	0·197
Grains	0·2	0·1, 0·3	0·002	0·2	0·0, 0·3	0·071	0·2	0·0, 0·4	0·064
Discret.	−0·2	−0·3, 0·0	0·014	−0·2	−0·3, −0·1	<0·001	−0·1	−0·3, 0·1	0·270
Lunch (serve/s)									
Fruit	0·3	0·1, 0·5	0·017	0·4	0·1, 0·6	0·005	0·2	0·0, 0·4	0·103
Vegetables	0·3	0·1, 0·5	0·001	**0·7**	**0·2, 1·2**	**0·010**	0·4	0·0, 0·7	0·041
Dairy products	0·1	0·0, 0·2	0·161	0·0	−0·1, 0·1	0·570	0·1	−0·1, 0·2	0·373
Proteins	0·0	−0·1, 0·1	0·832	0·1	−0·1, 0·4	0·223	0·2	0·0, 0·4	0·043
Grains	0·1	−0·1, 0·2	0·523	0·3	0·0, 0·5	0·024	0·1	−0·1, 0·3	0·174
Discret.	**−0·7**	**−0·9, −0·5**	**<0·001**	**−0·6**	**−0·8, −0·3**	**<0·001**	−0·4	−0·7, −0·2	0·001
Dinner (serve/s)									
Fruits	0·0	−0·2, 0·2	0·682	0·1	−0·1, 0·3	0·500	0·4	0·1, 0·7	0·004
Vegetables	**1·0**	**0·7, 1·2**	**<0·001**	**1·0**	**0·7, 1·4**	**<0·001**	**0·8**	**0·4, 1·3**	**0·001**
Dairy products	0·0	−0·1, 0·1	0·972	0·0	−0·1, 0·1	0·658	0·1	−0·1, 0·2	0·215
Proteins	0·2	0·0, 0·3	0·009	0·4	0·1, 0·6	0·002	0·3	0·1, 0·5	0·015
Grains	0·1	−0·2, 0·3	0·668	0·1	−0·2, 0·4	0·664	−0·3	−0·5, −0·1	0·007
Discret.	**−1·0**	**−1·3, −0·7**	**<0·001**	**−0·7**	**−1·0, −0·5**	**<0·001**	−0·2	−0·6, 0·2	0·261
Snack (serve/s)									
Fruits	0·4	0·3, 0·6	<0·001	0·4	0·1, 0·6	0·011	**0·5**	**0·1, 0·8**	**0·004**
Vegetables	0·2	−0·2, 0·5	0·319	0·2	−0·2, 0·6	0·288	0·4	−0·8, 1·7	0·489
Dairy products	0·2	0·0, 0·3	0·006	0·1	0·0, 0·3	0·078	0·1	0·0, 0·3	0·136
Proteins	0·1	0·0, 0·3	0·067	0·4	0·1, 0·6	0·005	0·3	0·1, 0·5	0·011
Grains	−0·2	−0·4, 0·0	0·051	−0·1	−0·3, 0·1	0·365	0·2	−0·1, 0·6	0·198
Discret.	**−1·0**	**−1·3, −0·7**	**<0·001**	**−1·1**	**−1·4, −0·8**	**<0·001**	**−0·6**	**−1·1, −0·2**	**0·007**
All eating occasions (serve/s)									
Fruits	**0·7**	**0·5, 0·9**	**<0·001**	**0·8**	**0·5, 1·1**	**<0·001**	**0·9**	**0·5, 1·3**	**<0·001**
Vegetables	**1·6**	**1·3, 1·9**	**<0·001**	**1·9**	**1·3, 2·4**	**<0·001**	**1·3**	**0·8, 1·8**	**<0·001**
Dairy products	**0·3**	**0·2, 0·5**	**<0·001**	0·3	0·1, 0·4	0·006	0·2	0·0, 0·4	0·062
Proteins	**0·5**	**0·3, 0·7**	**<0·001**	**0·7**	**0·4, 1·0**	**<0·001**	**0·7**	**0·4, 0·9**	**<0·001**
Grains	0·4	0·1, 0·7	0·017	0·3	−0·1, 0·8	0·177	0·1	−0·3, 0·5	0·687
Discret.	**−2·2**	**−2·6, −1·9**	**<0·001**	**−2·1**	**−2·5, −1·7**	**<0·001**	**−1·5**	**−2·0, −1·0**	**<0·001**

* Higher diet quality – the top tertile of dietary guidelines index score (0–130) which assessed adherence to the Australian Dietary Guidelines. Lower diet quality – bottom two tertiles of the score.

All results were weighted to be nationally representative of Australian population.

Dairy products – dairy product and alternatives foods.

Proteins – meat and alternatives foods.

Discret. – discretionary foods.

Bold – meaningful differences, where mean difference of food consumption (between those with high and low diet quality) are at least 0·5 serve with *P*-value < 0·05.

The weighted mean differences (95 % CI) of food group intakes among men with lower and higher diet quality are shown in Table 3 (for weighted mean serves, refer to Additional file 4). Men between 19 and 50 with higher diet quality consumed less discretionary foods at all eating occasions, particularly at dinner, and snacks, where the differences were more than one serves (M.D. for B, L, D, S: -0·3, -1·0, -1·3, -1·9 serves). Other differences were higher intake of vegetables at lunch and dinner (MD: 0·6 and 0·9 serves), more grain-based foods at breakfast (0·3 serves) and more meat and alternatives foods at snacks (0·5 serves). Similar to younger men, men between 51 and 70 with higher diet quality consumed less discretionary foods at all eating occasions, especially for snacks (MD: -1·3 serves). They also consumed more fruits and vegetables at lunch and dinner than men with lower diet quality. For snacks, men aged between 51 and 70 years with higher diet quality consumed more fruits and meat and alternatives foods (MD: 0·7 and 0·5 serves). No meaningful differences were found in consumption of discretionary foods at breakfast, lunch and snacks for men aged 71 years and over. Men aged 71 years and over with higher diet quality consumed more grains at breakfast (MD: 0·5 serves), more fruits at lunch (MD: 0·6 serves), more vegetables at dinner (MD: 1·2 serves) and fewer grain-based foods at snacks (-0·4 serves).

**Table 3.** Weighted mean differences in serves of food group consumption at eating occasions between Australian men with lower and higher level of diet quality*, stratified by age group (*n* 4245)

*n* (person)	19–50 years			51–70 years			71 years and over		
	2380			1334			531		
	Diff	95 % CI	*P*	Diff	95 % CI	*P*	Diff	95 % CI	*P*
Breakfast (serve/s)									
Fruits	0·4	0·1, 0·7	0·017	0·4	0·1, 0·7	0·005	0·6	0·0, 1·1	0·048
Vegetables	0·0	−0·5, 0·5	0·958	0·3	−0·4, 0·9	0·420	0·2	−0·5, 0·9	0·503
Dairy products	0·1	0·0, 0·2	0·011	0·3	0·2, 0·4	0·000	0·2	0·1, 0·3	0·005
Proteins	−0·1	−0·3, 0·1	0·280	0·0	−0·2, 0·2	0·925	0·2	−0·2, 0·7	0·277
Grains	0·3	0·1, 0·6	0·003	0·4	0·1, 0·6	0·011	**0·5**	**0·2, 0·8**	**0·001**
Discret.	−0·3	−0·4, −0·1	0·003	−0·4	−0·6, −0·1	0·001	−0·2	−0·4, 0·0	0·109
Lunch (serve/s)									
Fruits	0·3	0·1, 0·6	0·014	0·4	0·1, 0·6	0·008	**0·6**	**0·1, 1·1**	**0·022**
Vegetables	0·6	0·2, 0·9	0·004	**0·5**	**0·2, 0·9**	**0·004**	0·5	−0·3, 1·3	0·191
Dairy products	0·1	−0·1, 0·2	0·283	0·1	0·0, 0·2	0·178	0·2	0·0, 0·5	0·041
Proteins	0·1	0·0, 0·3	0·110	0·1	−0·1, 0·3	0·524	−0·1	−0·3, 0·2	0·647
Grains	−0·1	−0·3, 0·2	0·587	0·0	−0·3, 0·2	0·975	0·2	−0·1, 0·5	0·295
Discret.	**−1·0**	**−1·4, −0·7**	**<0·001**	**−0·6**	**−0·9, −0·3**	**<0·001**	−0·5	−1·1, 0·0	0·066
Dinner (serve/s)									
Fruits	0·4	0·2, 0·6	<0·001	0·4	0·1, 0·6	0·002	0·4	0·0, 0·8	0·071
Vegetables	**0·9**	**0·6, 1·3**	**<0·001**	**1·2**	**0·8, 1·6**	**<0·001**	**1·2**	**0·6, 1·7**	**<0·001**
Dairy products	0·0	−0·1, 0·2	0·471	−0·1	−0·3, 0·1	0·212	0·0	−0·1, 0·1	0·831
Proteins	0·1	−0·1, 0·3	0·333	0·2	−0·1, 0·5	0·201	0·1	−0·2, 0·4	0·377
Grains	0·2	−0·2, 0·5	0·335	0·1	−0·3, 0·4	0·679	−0·4	−0·8, 0·1	0·090
Discret.	**−1·3**	**−1·6, −0·9**	**0·001**	**−0·9**	**−1·4, −0·4**	**0·001**	**−0·8**	**−1·5, −0·2**	**0·010**
Snack (serve/s)									
Fruits	0·3	0·1, 0·6	0·016	**0·7**	**0·3, 1·1**	**0·001**	**0·5**	**0·1, 0·9**	**0·023**
Vegetables	0·2	−0·2, 0·7	0·304	−0·2	−1·0, 0·6	0·572	−0·4	−1·6, 0·8	0·475
Dairy products	0·1	0·0, 0·3	0·163	0·0	−0·2, 0·1	0·608	0·2	−0·1, 0·5	0·252
Proteins	**0·5**	**0·2, 0·7**	**<0·001**	0·4	0·1, 0·8	0·013	0·3	0·1, 0·6	0·016
Grains	−0·1	−0·4, 0·3	0·619	0·2	−0·2, 0·5	0·369	−0·4	−0·8, −0·1	0·005
Discret.	**−1·9**	**−2·2, −1·6**	**<0·001**	**−1·3**	**−1·8, −0·8**	**<0·001**	−0·5	−1·1, 0·1	0·127
Total (serve/s)									
Fruits	**1·0**	**0·7, 1·3**	**<0·001**	**1·3**	**1·0, 1·7**	**<0·001**	**1·1**	**0·6, 1·6**	**<0·001**
Vegetables	**1·5**	**1·1, 2·0**	**<0·001**	**1·9**	**1·4, 2·4**	**<0·001**	**1·6**	**0·9, 2·3**	**<0·001**
Dairy products	0·4	0·2, 0·6	<0·001	0·3	0·1, 0·6	0·006	0·4	0·2, 0·7	0·003
Proteins	**0·7**	**0·4, 1·0**	**<0·001**	**0·5**	**0·1, 0·8**	**0·008**	0·3	−0·1, 0·6	0·110
Grains	**0·8**	**0·3, 1·3**	**0·004**	**0·9**	**0·3, 1·4**	**0·005**	0·2	−0·4, 0·9	0·435
Discret.	**−3·8**	**−4·2, −3·3**	**< **0·001****	**−2·9**	**−3·4, −2·3**	**<0·001**	**−2·0**	**−2·9, −1·1**	**<0·001**

* Higher diet quality – the top tertile of dietary guidelines index score (0–130) which assessed adherence to the Australian Dietary Guidelines. Lower diet quality – bottom two tertiles of the score. All results were weighted to be nationally representative of Australian population.

Dairy products – dairy product and alternatives foods.

Proteins – meat and alternatives foods.

Discret. – discretionary foods.

Bold – meaningful differences, where mean difference of food consumption (between those with high and low diet quality) are at least 0·5 serve with *P*-value < 0·05.

### Differences in consumption stratified by education level

Tables 4 and 5 show the weighted mean differences (95 % CI) of food group intakes at eating occasions among men and women with higher and lower diet quality, by education level (for weighted mean serves, refer to Additional file 5 and 6). Stratification by education had little impact on the results, and the differences in food group consumptions found were consistent across education strata between men and women with lower and higher diet quality. We observed meaningful differences in intake of vegetables and discretionary foods at lunch and dinner, where women with higher diet quality consumed more vegetables and less discretionary food (L, D: 0·5 and -0·5 serve, 1·1 and -0·8 serves, all *P* values < 0·01). For snacks, meaningful differences in consumption were observed in fruits (0·5 serve (95 % CI (0·4, 0·6)) and discretionary foods (-1·0 serves (95 % CI (-1·2, -0·8)). Irrespective of their level of education, men with higher diet quality showed consistent dietary patterns where they consumed significantly fewer discretionary foods at lunch (-0·8 serves, 95 % CI (-0·9, -0·6)), dinner (-1·2 serves, 95 % CI (-1·4, -0·9)) and snacks (-1·5 serves, 95 % CI (-1·8, -1·3)). Additionally, these individuals incorporated more vegetables into their dinner (1·1 serves, 95 % CI (0·8, 1·3)) and higher fruit intake at snacks (0·6 serves, 95 % CI (0·4, 0·8)).

**Table 4.** Means differences* in serves of food group consumption at eating occasions between Australian women with lower and higher level of diet quality†, stratified by education level, adjusted for age (*n* 4809)

*n* (person)	Low level of education			Medium level of education			High level of Education		
	1534			1992			1283		
	Diff	95 % CI	*P*	Diff	95 % CI	*P*	Diff	95 % CI	*P*
Breakfast (serve/s)									
Fruits	0·2	0·1, 0·2	<0·001	0·2	0·1, 0·2	<0·001	0·2	0·1, 0·2	<0·001
Vegetables	0·1	0·0, 0·1	0·045	0·1	0·0, 0·1	0·045	0·1	0·0, 0·1	0·045
Dairy products	0·1	0·1, 0·2	<0·001	0·1	0·1, 0·2	<0·001	0·1	0·1, 0·2	<0·001
Proteins	0·0	−0·0, 0·1	0·067	0·0	−0·0, 0·1	0·067	0·0	−0·0, 0·1	0·067
Grains	0·2	0·1, 0·3	<0·001	0·2	0·1, 0·3	<0·001	0·2	0·1, 0·3	<0·001
Discret.	−0·2	−0·2, −0·1	<0·001	−0·2	−0·2, −0·1	<0·001	−0·2	−0·2, −0·1	<0·001
Lunch (serve/s)									
Fruit	0·1	0·1, 0·2	<0·001	0·1	0·1, 0·2	<0·001	0·1	0·1, 0·2	<0·001
Vegetables	**0·5**	**0·3, 0·7**	**<0·001**	**0·5**	**0·3, 0·7**	**<0·001**	**0·5**	**0·3, 0·7**	**<0·001**
Dairy products	0·0	−0·0, 0·1	0·884	0·0	−0·0, 0·1	0·884	0·0	−0·0, 0·1	0·884
Proteins	0·1	−0·0, 0·2	0·158	0·1	−0·0, 0·2	0·158	0·1	−0·0, 0·2	0·158
Grains	0·0	−0·1, 0·2	1·000	0·0	−0·1, 0·2	1·000	0·0	−0·1, 0·2	1·000
Discret.	**−0·5**	**−0·6, −0·4**	**<0·001**	**−0·5**	**−0·6, −0·4**	**<0·001**	**−0·5**	**−0·6, −0·4**	**<0·001**
Dinner (serve/s)									
Fruits	0·0	−0·0, 0·1	0·507	0·0	−0·0, 0·1	0·507	0·0	−0·0, 0·1	0·507
Vegetables	**1·1**	**0·8, 1·3**	**<0·001**	**1·1**	**0·8, 1·3**	**<0·001**	**1·1**	**0·8, 1·3**	**<0·001**
Dairy products	−0·0	−0·1, 0·0	0·515	−0·0	−0·1, 0·0	0·515	−0·0	−0·1, 0·0	0·515
Proteins	0·3	0·2, 0·4	<0·001	0·3	0·2, 0·4	0·000	0·3	0·2, 0·4	<0·001
Grains	−0·1	−0·3, 0·1	0·309	−0·1	−0·3, 0·1	0·309	−0·1	−0·3, 0·1	0·309
Discret.	**−0·8**	**−0·9, −0·6**	**<0·001**	**−0·8**	**−0·9, −0·6**	**<0·001**	**−0·8**	**−0·9, −0·6**	**<0·001**
Snack (serve/s)									
Fruits	**0·5**	**0·4, 0·6**	**<0·001**	**0·5**	**0·4, 0·6**	**<0·001**	**0·5**	**0·4, 0·6**	**<0·001**
Vegetables	0·0	−0·0, 0·1	0·609	0·0	−0·0, 0·1	0·609	0·0	−0·0, 0·1	0·609
Dairy products	0·1	0·0, 0·2	0·001	0·1	0·0, 0·2	0·001	0·1	0·0, 0·2	0·001
Proteins	0·1	0·1, 0·2	<0·001	0·1	0·1, 0·2	<0·001	0·1	0·1, 0·2	<0·001
Grains	−0·1	−0·2, 0·0	0·096	−0·1	−0·2, 0·0	0·096	−0·1	−0·2, 0·0	0·096
Discret.	**−1·0**	**−1·2, −0·8**	**<0·001**	**−1·0**	**−1·2, −0·8**	**<0·001**	**−1·0**	**−1·2, −0·8**	**<0·001**

* Difference in serves between lower and higher diet quality participants, by comparing marginal means. Bonferroni-adjusted 95 % confidence interval and p value are reported.

† Higher diet quality – the top tertile of dietary guidelines index score (0–130) which assessed adherence to the Australian Dietary Guidelines. Lower diet quality – bottom two tertiles of the score.

Dairy product – dairy product and alternatives foods.

Proteins – meat and alternatives foods.

Discret. – discretionary foods.

Bold – meaningful differences, where mean difference of food consumption (between those with high and low diet quality) are at least 0·5 serve with *P*-value < 0·05.

**Table 5.** Means differences* in serves of food group consumption at eating occasions between Australian men with lower and higher level of diet quality†, stratified by education level, adjusted for age (*n* 4245)

*n* (person)	Low level of education			Medium level of education			High level of education		
	1039			2198			1008		
	Diff	95 % CI	*P*	Diff	95 % CI	*P*	Diff	95 % CI	*P*
Breakfast (serve/s)									
Fruits	0·4	0·2, 0·5	<0·001	0·4	0·2, 0·5	<0·001	0·4	0·2, 0·5	<0·001
Vegetables	0·1	−0·0, 0·1	0·329	0·1	−0·0, 0·1	0·329	0·1	−0·0, 0·1	0·329
Dairy products	0·2	0·1, 0·3	<0·001	0·2	0·1, 0·3	<0·001	0·2	0·1, 0·3	<0·001
Proteins	0·0	−0·1, 0·1	1·000	0·0	−0·1, 0·1	1·000	0·0	−0·1, 0·1	1·000
Grains	0·4	0·3, 0·6	<0·001	0·4	0·3, 0·6	<0·001	0·4	0·3, 0·6	<0·001
Discret.	−0·3	−0·4, −0·2	<0·001	−0·3	−0·4, −0·2	<0·001	−0·3	−0·4, −0·2	<0·001
Lunch (serve/s)									
Fruits	0·2	0·1, 0·3	<0·001	0·2	0·1, 0·3	<0·001	0·2	0·1, 0·3	<0·001
Vegetables	0·4	0·1, 0·7	0·001	0·4	0·1, 0·7	0·001	0·4	0·1, 0·7	0·001
Dairy products	0·1	0·0, 0·1	0·043	0·1	0·0, 0·1	0·043	0·1	0·0, 0·1	0·043
Proteins	0·1	−0·0, 0·2	0·277	0·1	−0·0, 0·2	0·277	0·1	−0·0, 0·2	0·277
Grains	−0·0	−0·3, 0·2	1·000	−0·0	−0·3, 0·2	1·000	−0·0	−0·3, 0·2	1·000
Discret.	**−0·8**	**−0·9, −0·6**	**<0·001**	**−0·8**	**−0·9, −0·6**	**<0·001**	**−0·8**	**−0·9, −0·6**	**<0·001**
Dinner (serve/s)									
Fruits	0·2	0·1, 0·2	<0·001	0·2	0·1, 0·2	<0·001	0·2	0·1, 0·2	<0·001
Vegetables	**1·1**	**0·8, 1·3**	**<0·001**	**1·1**	**0·8, 1·3**	**<0·001**	**1·1**	**0·8, 1·3**	**<0·001**
Dairy products	0·0	−0·1, 0·1	1·000	0·0	−0·1, 0·1	1·000	0·0	−0·1, 0·1	1·000
Proteins	0·1	−0·0, 0·3	0·171	0·1	−0·0, 0·3	0·171	0·1	−0·0, 0·3	0·171
Grains	−0·1	−0·3, 0·2	1·000	−0·1	−0·3, 0·2	1·000	−0·1	−0·3, 0·2	1·000
Discret.	**−1·2**	**−1·4, −0·9**	**<0·001**	**−1·2**	**−1·4, −0·9**	**<0·001**	**−1·2**	**−1·4, −0·9**	**<0·001**
Snack (serve/s)									
Fruits	**0·6**	**0·4, 0·8**	**<0·001**	**0·6**	**0·4, 0·8**	**<0·001**	**0·6**	**0·4, 0·8**	**<0·001**
Vegetables	0·0	−0·1, 0·1	1·000	0·0	−0·1, 0·1	1·000	0·0	−0·1, 0·1	1·000
Dairy products	0·1	−0·0, 0·2	0·081	0·1	−0·0, 0·2	0·081	0·1	−0·0, 0·2	0·081
Proteins	0·3	0·1, 0·4	<0·001	0·3	0·1, 0·4	<0·001	0·3	0·1, 0·4	<0·001
Grains	−0·0	−0·2, 0·2	1·000	−0·0	−0·2, 0·2	1·000	−0·0	−0·2, 0·2	1·000
Discret.	**−1·5**	**−1·8, −1·3**	**<0·001**	**−1·5**	**−1·8, −1·3**	**<0·001**	**−1·5**	**−1·8, −1·3**	**<0·001**

* Difference in serves between low and high diet quality participants, by comparing marginal means. Bonferroni-adjusted 95 % CIl and *P* value are reported.

† Higher diet quality – the top tertile of dietary guidelines index score (0–130) which assessed adherence to the Australian Dietary Guidelines. Lower diet quality – bottom two tertiles of the score.

Dairy products – dairy product and alternatives foods.

Proteins – meat and alternatives foods.

Discret. – discretionary foods.

Bold – meaningful differences, where mean difference of food consumption (between those with high and low diet quality) are at least 0·5 serve with *P*-value < 0·05.

## Discussion

This study examined differences in foods and beverages consumed at eating occasions across levels of diet quality and across key socio-demographic factors and found that the intake of vegetables and discretionary foods at lunch, dinner and snacks was meaningfully different between adults with higher and lower diet quality. The study found that adults with higher diet quality consumed fewer discretionary foods at snacks, more vegetables at dinner and had fewer eating occasions that contained discretionary foods compared with those with lower diet quality. When age groups were adjusted, those with lower diet quality consistently consumed more discretionary foods at lunch, dinner and snacks, regardless of their education level. These findings on food group consumption at eating occasions demonstrate how the current eating patterns of Australians contribute to better diet quality and identify dinner and snacks as opportunities to increase vegetable intake and reduce discretionary food intake, respectively. They are relevant to the translation of messages around consumption of food groups at eating occasions.

Findings from the present study found that discretionary food intake differed significantly at snacks between adults with higher and lower diet quality, except for those above 70 years of age. We found that adults with lower diet quality consumed more servings of discretionary foods at snacks. In addition to consuming a greater quantity, those with lower diet quality also had a greater number of eating occasions where consumption of discretionary foods occurred. This is consistent with previous research that showed higher discretionary food consumption was associated with lower diet quality^(23,24)^. Therefore, recommendations regarding discretionary foods may have two approaches: reduce the frequency of snacks with discretionary foods or reduce the amount of discretionary food consumed when snacking occurs. An expert collaboration across several fields of research found that strategically designed snacking can be a useful tool for adults to meet their daily dietary needs^(25)^. In addition, the research concluded that snacking could assist in making healthy dietary choices when done thoughtfully and responsibly. Tailored strategies addressing discretionary food and beverage intake through portion control, frequency reduction or variety reduction were also found to lower energy intake and enhance diet quality^(26)^, suggesting that interventions need to customise messages to individuals’ dietary habits. Therefore, snacks are an important occasion to target for reducing and replacing discretionary foods (with fruits and vegetables). Results from a randomised controlled trial have showed that interventions aimed at increasing fruit and vegetable consumption among socio-economically disadvantaged individuals were effective in reducing their consumption of discretionary foods^(27)^. Additionally, a study investigating food swaps showed that substitutions in the diet are feasible and could form the basis of a dietary strategy to improve overall quality. They found that fruit consumption increased when swapped with discretionary foods while there was limited change in vegetable^(28)^. This recommendation is in line with a scoping review that aimed to identify dietary intervention strategies to reduce intake of discretionary choices, where restricting portion size reduced energy intake consistently in acute settings and substituting discretionary choices for high fibre snacks, fruit or low/no-energy beverages were identified as helpful strategies^(29)^.

Overall, adults who achieved higher diet quality consumed more vegetables at the dinner eating occasion. We also found that those with lower diet quality had fewer occasions where vegetable consumption (at lunch and dinner) occurred. It has been shown in another study that preparing meals with vegetables daily was associated with higher diet quality^(30)^. Increasing vegetable consumption is critical to improve overall health in the long term^(31)^, and, in the short term, it also improves diet quality and energy density^(32)^. Therefore, strategies for increasing vegetable consumption may emphasise the dinner meal and encourage those who do not eat vegetables at dinner to try adding vegetables, while those already consuming vegetables at dinner could be encouraged to increase their portion or serving size. Similar recommendations were implemented by a study that used mobile apps to increase vegetable consumption and found that vegetable intakes increased by half a serving^(33)^. In a different vein, while our results showed a meaningful difference in vegetable consumption (for women 19–50 years old) at breakfast, indicating its potential as a valuable dietary strategy, implementing such changes will require significant shifts in eating habits, preferences and cooking skills. Nevertheless, it presents a potential area for future research to investigate the feasibility and acceptability of such strategies.

Adults who achieved higher diet quality also consistently consumed more meat and alternatives foods at snacks. This observation suggests that consuming meat and alternatives foods at snacks may be another strategy for improving diet quality. However, another study has highlighted that Australian adults consumed little meat and alternatives foods at snack, with only 12–13 % of men and women consuming foods such as cheeses, milk and processed meat^(34)^. Further research is warranted to explore the specific protein sources and their nutritional contributions during snack periods among individuals with varying diet quality scores, as this could inform targeted dietary interventions and recommendations. This is important as the range of food items in this category may not always be healthy, and intervention needs to promote non-discretionary versions.

Food consumption patterns indicate that some foods are rarely consumed at certain eating occasions. We found little variation in food group consumption at breakfast between those with lower and higher diet quality. For example, most Australian adults do not consume vegetables and meats and alternatives at breakfast or snack time regardless of overall diet quality; however, fruit, grains and dairy products and alternatives foods are commonly included. This is consistent with previous research on breakfast consumption among Australians^(35)^. With respect to lunch, we found that grains, meats and alternatives and vegetables are more commonly consumed, while at dinner, grains, meats and alternatives and vegetables are the most common, and for snacks, fruit, dairy product and alternatives and discretionary foods, respectively. Certain food groups are more favoured to certain eating occasions, which shows how Australians consume food at mealtime, even those on a healthier diet. The types of eating patterns that work best for nutrition promotion messages can be derived from knowledge of the most likely foods to be consumed. For example, messages urging Australians to eat more vegetables at breakfast may be more challenging to adopt as this is not how Australians currently achieve a healthier diet. Further research is needed to examine barriers to consuming these foods at individual eating occasions.

Collecting a wide range of potential determinants when eating occasions occur is important when analysing food intake. After adjusting for age group, we found that differences in vegetable and discretionary food consumption remained significant at lunch, dinner and snack across all education levels. A study in Norwegian adults also found that the food group that was the main contributor to a meal did not change when studying subgroups with different education levels^(36)^. In a recent nationwide survey of Australian young adults, it was observed that individuals with stronger social support networks and greater food security exhibited higher levels of diet quality^(37)^. These social determinants provided a more comprehensive explanation for the disparities observed in food consumption during eating occasions. Other barriers that further explained the differences in diets included influences from family and peers, expected consumption of unhealthy foods in certain situations, presence and cost of discretionary foods^(30,38)^. If this contextual information was collected when consumption occurred, it would provide a more comprehensive picture of Australians’ eating patterns with varying diet quality.

### Strengths and limitations

This study had several strengths and limitations. Strengths included the use of a large national sample of Australian adults. Second, all estimates of food consumption were examined by sex and age groups reflective of the ADG and enables comparison to other studies and ease of interpretation. Finally, the DGI score has been evaluated as a useful measure of diet quality in previous studies examining its association with nutrient intake and health outcomes^(19)^.

This study has limitations, which should be acknowledged. A potential limitation is the use of a single 24-h recall to estimate food consumption, which involves some measurement error and does not allow estimation of day-to-day variability in individual food intakes^(39)^. However, our analysis used the first day of dietary recall, which was conducted across all days of the week and all seasons of the year, which allowed us to maintain the national representativeness of the sample and estimate average usual intakes of population groups. The analysis did not account for weekday-weekend variations, potentially affecting mean differences in consumption between populations with higher and lower diet quality. Nonetheless, when comparing food group consumption between weekdays and weekends, stratified by gender (data not shown), we observed minimal differences. The survey was conducted between 2011 and 2012, possibly not reflecting current Australian food consumption trends; nevertheless, it is the most recent available data. The classification of meals or snacks in this study relied on participant identification of eating occasions. Thus, the research must decide how to categorise ambiguous eating occasions, such as supper, as a meal or snack. Furthermore, all instances outside of main meals were classified as snacks without accounting for the time of day of the eating occasions. Considering that foods consumed during snacks may vary by time of day^(40)^, interpreting our findings requires caution. Additionally, the tertile approach to identify higher and lower diet quality was not stratified, resulting in a higher representation of women in the higher diet quality population. Furthermore, this analysis compared the top tertile with the lower two tertiles, examining extreme tertile groups (Tertile 1 *v*. Tertile 3) might have revealed even more pronounced differences in eating patterns. Lastly, the results cannot be generalised internationally since consumption varies from country to country. Again, caution is needed when interpreting the results of the survey and extrapolating the findings to apply to the current diets of Australians or those from other countries. Still, the methods used may guide future studies, and results may act as a comparison to future data.

### Future research

Several future directions can be considered from this research, including potential methodological advances concerning examining food intake at eating occasions. While this study included education level as a potential explanation for the variations in consumption, other variables may better explain changes in consumption at the level of eating occasion. Understanding a broader range of determinants of food intake at eating occasions beyond socio-demographic factors is essential^(41)^. Contextual factors such as the location of where consumption occurs and the presence of others at consumption may further provide a more comprehensive picture of dietary behaviour. Unfortunately, these variables were not available in the national survey. Furthermore, other socio-demographic factors available in the National Nutrition and Physical Activity Survey, like ethnicity, were not adequately represented, which may be a consideration for future national surveys.

### Conclusions

This study found that the intake of vegetables and discretionary foods at lunch, dinner and snacks was meaningfully different between adults with higher and lower diet quality. When stratified by age and sex, adults with higher diet quality consumed less discretionary foods at snack events and more vegetables at dinner. After stratifying by education levels, similar dietary patterns were observed, suggesting that other factors may be involved in differences in intake during eating occasions between those with higher and lower adherence to dietary guidelines. Consequently, strategies for increasing vegetable consumption may emphasise the dinner meal and encourage those who do not eat vegetables at dinner to try adding them, in addition to encouraging those already eating vegetables to consume a larger portion. Like dinner, snacks are an important opportunity to reduce and replace discretionary foods with fruits and vegetables. On the other hand, contextual information surrounding eating occasions is needed to better understand the population’s dietary patterns and design future interventions. An improved understanding of what drives dietary decisions during different eating occasions is essential for promoting healthy eating habits among different populations. Meal-specific advice beyond demographic characteristics is may be useful for encouraging positive and feasible dietary changes that may lead to increased adherence to dietary guidelines.

## Supporting information

Tran et al. supplementary material 1 Tran et al. supplementary material

Tran et al. supplementary material 2 Tran et al. supplementary material

Tran et al. supplementary material 3 Tran et al. supplementary material

Tran et al. supplementary material 4 Tran et al. supplementary material

Tran et al. supplementary material 5 Tran et al. supplementary material

Tran et al. supplementary material 6 Tran et al. supplementary material

## Supplementary material

For supplementary material/s referred to in this article, please visit https://doi.org/10.1017/S0007114523002325
