# Supplementary material for: Achieving high diet quality at eating occasions: findings from a nationally representative study of Australian adults
Source: Br J Nutr. 2023 Oct 19;131(5):868–79. doi: 10.1017/S0007114523002325 (PMC10864991; doi:10.1017/S0007114523002325)
Supplement: Tran et al. supplementary material 1 — Tran et al. supplementary material [file S0007114523002325sup001.docx]

**Additional file 1. Methods of adapting the Australian Dietary Guidelines to 24-hour recall data.**

| Dietary Guideline | Component and Description | Criteria for Maximum Score ^1^ | Criteria for Minimum Score | Maximum Score | Method of Score Computation for 24-her recall |
| --- | --- | --- | --- | --- | --- |
| Guidelines for adequate intake | | | | |  |
| 1. Enjoy a wide variety of nutritious foods | Food variety ^2^: proportion of food from each of the 5 core food groups eaten at least one serve per week | 100% | 0% | 10 | Score (out of 10) were divided into 5 categories: Vegetables, Fruits, Proteins, Dairy, and Wholegrains.    Vegetable Score:  To calculate the Vegetable score, we need to determine whether an individual consume more than 30 grams of beverages or 15 grams of food from each vegetable category. Then, calculate the Vegetable score as the average of five different vegetable categories:  - Green Brassica Vegetables  - Orange Vegetables  - Starchy Vegetables  - Legumes (considered as a vegetable)  - Other Vegetables  Sum up the quantities consumed from each of these categories and divide the total by 5 to get the Vegetables score. This score represents how well the individual meet the recommended consumption levels for these various vegetable categories.  Fruit Score:  To calculate the Fruit score, we need to determine whether an individual consume more than 30 grams of beverages or 15 grams of food from each fruit category. Then, calculate the Fruit score as the average of six different fruit categories:   - Fruit juice - Stone fruits - Berry fruits - Citrus fruits - Tropical fruits - Other fruits   Sum up the quantities consumed from each of these categories and divide the total by 6 to get the Fruit score. This score represents how well an individual meet the recommended consumption levels for these various fruit categories.  Proteins Score:  To calculate the Proteins Score, we first determine whether an individual consume more than 30 grams of meat-based beverages or 15 grams of meat-based foods from the lean meat category or suitable alternatives. Then, you calculate the Protein score as the average of four different protein categories:   - Red Meat - Poultry - Fish - Meat Alternatives   Sum up the quantities consumed from each of these protein categories and divide the total by 4 to get Proteins score. This score represents how well an individual meet the recommended consumption levels for these various protein sources, considering both meat and suitable alternatives.  Dairy Score:  To calculate the Dairy Score, we first determine whether an individual consume more than 30 grams of dairy-based beverages or 15 grams of dairy-based foods or suitable alternatives. Then, calculate the score as the average of three different dairy product categories:   - Milk - Yogurt - Cheese   Sum up the quantities consumed from each of these dairy categories and divide the total by 3 to get the Dairy Score. This score represents how well an individual meet the recommended consumption levels for various dairy products and suitable alternatives.  Wholegrain Cereal Score:  To calculate the Wholegrain Cereal Score, we first determine whether an individual consume more than 30 grams of cereal-based beverages or 15 grams of cereal-based foods. Then, calculate the score as the average of three different whole grain cereal categories:   - Wholegrain Bread - Wholegrain Grains - Wholegrain Cereal   Sum up the quantities consumed from each of these whole grain cereal categories and divide the total by 3 to get the Whole Grain Cereal Score. This score represents how well an individual meet the recommended consumption levels for various wholegrain cereal products.  Add all scores together. |
| 2. Plenty of vegetables | Total vegetable intake: servings of vegetables per day | 19–50 y: M ≥ 6, F ≥ 5 | 0 | 10 | Used ADG Classification Code 20 to calculate servings.  Handling Missing Values (assumed that for Day 1, they did not consume any products): For individuals with missing values, they are assigned a score of 5. |
|  |  | 51–70 y: M ≥ 5.5, F ≥ 5 |  |  |  |
|  |  | > 70 y: M ≥ 5, F ≥ 5 |  |  |  |
| 3. Fruit | Total fruit intake: servings of fruit per day | ≥2 | 0 | 10 | Used ADG Classification Code 30 to calculate servings.  Handling Missing Values (assumed that for Day 1, they did not consume any products): For individuals with missing values, they are assigned a score of 5. |
| 4. Grain (cereal) foods | Total cereal intake: servings of grains per day | 19–50 y: M ≥ 6, F ≥ 6 | 0 | 5 | Used ADG Classification Code 10 to calculate servings.  Handling Missing Values (assumed that for Day 1, they did not consume any products): For individuals with missing values, they are assigned a score of 2.5. |
|  |  | 51–70 y: M ≥ 6, F ≥ 4 |  |  |  |
|  |  | >70 y: M ≥ 4.5, F ≥ 3 |  |  |  |
|  | Mostly wholegrain or high fibre cereals: Type of bread usually consumed | Wholemeal bread | White bread | 5 | This scoring system is used to assess how well individuals meet the recommended wholegrain consumption criteria, with a score of 5 indicating that they consume at least 50% whole grain bread, and lower scores reflecting varying levels of whole grain consumption.  Rule: If more than or equal to 50% of the bread consumed is wholegrain (as opposed to white bread), it earns a score of 5 points.  Using the ADG (Australian Dietary Guidelines) Classification Code 101 to determine whole grain consumption in servings.  For individuals with missing or no consumption data on Day 1, and these individuals consumed cereal, they are treated as having no consumption of wholegrain because there's no data indicating otherwise.  First, calculate the ratio of whole grain consumption to total gram consumption, where Wholegrain/Total Grain Consumption = Ratio.  Score Calculation: Depending on the value of the ratio, you assign a score as follows:  When the Ratio is less than 0.5, the score is calculated as Ratio * 10.  When the Ratio is equal to or greater than 0.5, the score is a fixed value of 5.  Handling Missing Ratios (assumed that for Day 1, they did not consume any grain products): For individuals with missing ratio values, they are assigned a score of 2.5. |
| 5. Lean meat and poultry, fish, eggs, nuts and seeds, and legumes/beans | Total meat and alternative: servings per day | 19–50 y: M ≥ 3, F ≥ 2.5 | 0 | 5 | Used ADG Classification Code 50 to calculate servings.  Handling Missing Values (assumed that for Day 1, they did not consume any protein products): For individuals with missing values, they are assigned a score of 2.5. |
|  |  | 51–70 y: M ≥ 2.5, F ≥ 2 |  |  |  |
|  |  | >70 y: M ≥ 2.5, F ≥ 2 |  |  |  |
|  | Lean meat: proportion of lean meats and alternatives to total meat and alternatives per day | 100% | 0% | 5 | Used ADG Classification Code:   - 501, 503, 505, 506, 507, 508   Rule: If more than or equal to 50% of proteins consumed is unprocessed meats, poultry, fish, eggs, tofu, nuts, seeds, legumes/beans (as opposed to not met ADG criteria), it earns a score of 5 points.  Code:  Good proteins/Total Protein consumption = Ratio  When Ratio is less than 0.5, score = Ratio*10  When Ratio is equal or more than 0.5, score = 5. |
| 6. Milk, yoghurt, cheese and/or their alternatives ^3^ | Total dairy and alternative: servings per day | 19–50 y: M ≥ 2.5, F ≥ 2.5 | 0 | 10 | Used ADG Classification Code 40.  Handling Missing Values (assumed that for Day 1, they did not consume any products): For individuals with missing values, they are assigned a score of 5. |
|  |  | 51–70 y: M ≥ 2.5, F ≥ 4 |  |  |  |
|  |  | >70 y: M ≥ 3.5, F ≥ 4 |  |  |  |
| 7. Drink plenty of water | Total beverage intake ^4^: servings per day | M ≥ 10; F ≥ 8 | 0 | 5 | Used ADG Classification Code 60.  Handling Missing Values (assumed that for Day 1, they did not consume any products): For individuals with missing values, they are assigned a score of 2.5. |
|  | Water ^5^: proportion of water to total beverage intake per day | ≥50% | 0% | 5 | RULE: More than 50% fluid is water (thee digits food code: 117)  Missing/ no consumption = 1415. Assumed no consumption of water.  Code:  Water/Total beverages consumption = Ratio  When Ratio is less than 0.5, score = Ratio*10  When Ratio is equal or more than 0.5, score = 5. |
| Guidelines to limit or moderate intake | | | | |  |
| 8. Limit intake of foods containing saturated fat, added salt, added sugars and alcohol | Limit discretionary foods | M ≤ 3; F ≤ 2.5 | M > 3; F > 2.5 | 10 | Used ABS provided for flagged discretionary items.  Missing have score of 5. |
| 9. Limit intake of foods high in saturated fat | Trim meat: trimming fat from meat | Usually | Never or rarely | 5 | Generate ratio of trimmed (fully & semi) meat to total meat intake.  INCLUDE: beef, lamb, pork, veal, bacon, mutton, meat alternative |
|  | Choose reduced-fat milk: type of milk usually consumed | Skim, low or reduced fat milk. | Whole milk | 5 | Dairy Fat Score Calculation:  The Dairy Fat Score is calculated based on the consumption of lower fat (LF) and medium fat (MF) dairy foods relative to the total dairy consumption. This calculation is performed using ADG Classification Codes 402 and 403.  Score Calculation:  First, calculate the ratio of lower fat (LF) dairy consumption to the total dairy consumption.  Based on the calculated ratio, assign a score as follows:  When the Ratio is less than 0.5, the score is calculated as Ratio * 10.  When the Ratio is equal to or greater than 0.5, the score is a fixed value of 5.  This scoring system assesses how well individuals meet the recommended consumption of lower fat and medium fat dairy foods relative to their total dairy consumption. Scores vary based on the ratio of lower fat dairy consumption to total dairy consumption, with a higher score indicating a greater proportion of lower fat dairy in the diet. |
| 10. Small allowance of unsaturated oils, fats or spreads | Unsaturated spreads and oils: servings per day | 19–50 y: M ≤ 4, F ≤ 2 | M > 4; F > 2 | 10 | Used ADG classification code 70.  Handling Missing Values (assumed that for Day 1, they did not consume any products): For individuals with missing values, they are assigned a score of 5. |
|  |  | 51–70 y: M ≤ 4, F ≤ 2 |  |  |  |
|  |  | >70 y: M ≤ 2, F ≤ 2 |  |  |  |
| 11. Limit intake of foods and drinks containing added salt | Salt use: salt added during cooking | Never or rarely | Usually | 5 | DIETQ12  Rule:  If "DIETQ12" is "Very often", the value is 0.  If "DIETQ12" is "Occasionally", the value is 1.25.  If "DIETQ12" is "Not known", the value is 2.5.  If "DIETQ12" is "Rarely", the value is 3.75.  If "DIETQ12" is "Not used", the value is 5. |
|  | Salt use: salt added during the meal | Never or rarely | Usually | 5 | DIETQ14  Rule:  If "DIETQ14" is "Very often", the value is 0.  If "DIETQ14" is "Occasionally", the value is 1.25.  If "DIETQ14" is "Not known", the value is 2.5.  If "DIETQ14" is "Rarely", the value is 3.75.  If "DIETQ14" is "Not used", the value is 5. |
| 12. Limit intake of foods and drinks containing added sugars | Limit extra sugar ^6^: servings per day | M ≤ 1.5; F ≤ 1.25 | M > 1.5; F > 1.25 | 10 | Calculation of Added Sugar Serves:  To calculate the serves of added sugar, specific food codes are used. These food codes fall into three categories based on the number of digits: 5 digits, 3 digits, and 2 digits.  5 Digits Food Codes:  "12305", "12306", "13402", "13403", "13601", "13602", "13603", "13605"  3 Digits Food Codes:  "125", "131", "133", "195", "197", "204"  2 Digits Food Codes:  "11", "27", "28"  In all cases, the calculation considers 1 serve of discretionary food to be equivalent to 600 kilojoules (kJ) and includes the presence of fiber in the food. |
| 13. If you choose to drink alcohol, limit intake | Limit alcohol: servings per day | ≤2 | >2 | 10 | Alcohol Consumption Score Calculation:  For food code 29, which is associated with alcohol consumption, the scoring is based on the amount of alcohol consumed:  Standard Drink: 10 grams of alcohol is considered a standard drink.  Maximum Score: The maximum score is given when an individual consumes no more than 2 standard drinks per day, which is equivalent to 20 grams of alcohol. In this case, they receive a score of 10.  Missing/No Alcohol Consumption: If there is missing data or an individual did not consume any alcohol, they are assigned a score of 10. This score is given to indicate that they have not consumed alcohol or that there's insufficient data to determine their alcohol consumption.  Scoring Range: The scoring for this item can only be 0 (indicating no alcohol consumption or exceeding the recommended limit) or 10 (indicating adherence to the recommended limit of 2 standard drinks per day or less). |
