# Supplementary material for: Achieving high diet quality at eating occasions: findings from a nationally representative study of Australian adults
Source: Br J Nutr. 2023 Oct 19;131(5):868–79. doi: 10.1017/S0007114523002325 (PMC10864991; doi:10.1017/S0007114523002325)
Supplement: Tran et al. supplementary material 2 — Tran et al. supplementary material [file S0007114523002325sup002.docx]

**Additional file 2. Frequency and weighted proportion of eating occasions containing the five food group foods and discretionary foods among consumers and non-consumers, by level of diet quality.^1^**

|  | **Lower Diet Quality** | | | | **Higher Diet Quality** | | | |
| --- | --- | --- | --- | --- | --- | --- | --- | --- |
| **Person(s)** | **n** | | **%** | | **n** | | **%** | |
|  | 6037 | | 66.8 | | 3017 | | 33.2 | |
|  | **Consumer** | | **Non-consumer** | | **Consumer** | | **Non-consumer** | |
| **Eating occasions** | **n** | **%** | **n** | **%** | **n** | **%** | **n** | **%** |
| **Breakfast** | | | | | | | | |
| Fruit | 2240 | 42 | 2972 | 58 | 1628 | 54 | 1258 | 46 |
| Veg | 713 | 14 | 4499 | 86 | 418 | 16 | 2468 | 84 |
| Dairy | 3928 | 74 | 1284 | 26 | 2370 | 82 | 516 | 18 |
| Proteins | 1628 | 32 | 3584 | 68 | 977 | 36 | 1909 | 64 |
| Grains | 4480 | 86 | 732 | 14 | 2600 | 90 | 286 | 10 |
| Discretionary | 3323 | 63 | 1889 | 37 | 1439 | 51 | 1447 | 49 |
| **Lunch** | | | | | | | | |
| Fruit | 1647 | 33 | 3499 | 67 | 1020 | 37 | 1806 | 63 |
| Veg | 3522 | 69 | 1624 | 31 | 2205 | 77 | 621 | 23 |
| Dairy | 2550 | 48 | 2596 | 52 | 1431 | 49 | 1395 | 51 |
| Proteins | 3858 | 75 | 1288 | 25 | 2134 | 77 | 692 | 23 |
| Grains | 4312 | 85 | 834 | 15 | 2330 | 83 | 496 | 17 |
| Discretionary | 3448 | 67 | 1698 | 33 | 1473 | 53 | 1353 | 47 |
| **Dinner** | | | | | | | | |
| Fruit | 1854 | 32 | 3846 | 68 | 1082 | 36 | 1874 | 64 |
| Veg | 4976 | 87 | 724 | 13 | 2747 | 93 | 209 | 7 |
| Dairy | 2904 | 50 | 2796 | 50 | 1421 | 48 | 1535 | 52 |
| Proteins | 4956 | 87 | 744 | 13 | 2670 | 90 | 286 | 10 |
| Grains | 4225 | 76 | 1475 | 24 | 2075 | 70 | 881 | 30 |
| Discretionary | 3959 | 69 | 1741 | 31 | 1545 | 51 | 1411 | 49 |
| **Snack** | | | | | | | | |
| Fruit | 2815 | 48 | 3049 | 52 | 2028 | 69 | 926 | 31 |
| Veg | 1140 | 21 | 4724 | 79 | 593 | 21 | 2361 | 79 |
| Dairy | 4202 | 70 | 1662 | 30 | 2215 | 74 | 739 | 26 |
| Proteins | 2055 | 36 | 3809 | 64 | 1228 | 43 | 1726 | 57 |
| Grains | 3210 | 56 | 2654 | 44 | 1581 | 55 | 1373 | 45 |
| Discretionary | 4950 | 85 | 914 | 15 | 2168 | 75 | 786 | 25 |

^1^Consumer/non-consumer indicates whether the food group was consumed/not consumed at the specified eating occasion.
