# Supplementary material for: Achieving high diet quality at eating occasions: findings from a nationally representative study of Australian adults
Source: Br J Nutr. 2023 Oct 19;131(5):868–79. doi: 10.1017/S0007114523002325 (PMC10864991; doi:10.1017/S0007114523002325)
Supplement: Tran et al. supplementary material 3 — Tran et al. supplementary material [file S0007114523002325sup003.docx]

**Additional file 3. Weighted means^1^ in serves of food group consumption at eating occasions between Australian women with lower and higher level of diet quality^2^, stratified by age group. (n = 4809)**

|  |  | | | |  | | | |  | | | |
| --- | --- | --- | --- | --- | --- | --- | --- | --- | --- | --- | --- | --- |
|  | **19-50 years** | | | | **51-70 years** | | | | **71 years and over** | | | |
| n (person) | 2512 | | | | 1556 | | | | 741 | | | |
|  | **Low**er  DQ | | **High**er  **DQ** | | **Low**er  DQ | | **High**er  **DQ** | | **Low**er  DQ | | **High**er  **DQ** | |
| n (person) | 1567 | | 945 | | 1011 | | 545 | | 441 | | 300 | |
|  | **Mean** | **95%**  **CI** | **Mean** | **95%**  **CI** | **Mean** | **95%**  **CI** | **Mean** | **95%**  **CI** | **Mean** | **95%**  **CI** | **Mean** | **95%**  **CI** |
| Breakfast (serve/s) | | | | | | | | | | | | |
| Fruit  Vegetables  Dairy  Proteins  Grains  Discret. | 0.8  0.9  0.6  0.6  1.5  0.8 | 0.7, 0.9  0.7, 1.1  0.6, 0.7  0.5, 0.6  1.5, 1.6  0.7, 0.9 | 0.9  1.4  0.7  0.6  1.7  0.6 | 0.8, 0.9  1.1, 1.8  0.7, 0.8  0.5, 0.6  1.6, 1.8  0.5, 0.7 | 0.8  1.0  0.5  0.5  1.6  0.6 | 0.7, 0.9  0.7, 1.4  0.5, 0.6  0.4, 0.6  1.4, 1.7  0.5, 0.7 | 1.0  1.2  0.7  0.6  1.7  0.4 | 0.8, 1.1  0.8, 1.6  0.6, 0.7  0.5, 0.7  1.6, 1.9  0.3, 0.4 | 0.7  0.7  0.5  0.3  1.5  0.6 | 0.6, 0.8  0.3, 1.0  0.4, 0.6  0.2, 0.4  1.4, 1.6  0.4, 0.7 | 1.0  1.2  0.6  0.5  1.7  0.5 | 0.8, 1.2  0.5, 1.8  0.5, 0.7 0.3, 0.6  1.5, 1.9  0.4, 0.6 |
| Lunch (serve/s) | | | | | | | | | | | | |
| Fruit  Vegetables  Dairy  Proteins  Grains  Discret. | 0.7  1.2  0.7  0.9  1.8  1.6 | 0.6, 0.8  1.1, 1.3  0.6, 0.7  0.9, 1.0  1.7, 2.0  1.5, 1.8 | 1.0  1.5  0.7  1.0  1.9  1.0 | 0.8, 1.2  1.4, 1.6  0.7, 0.8  0.9, 1  1.8, 2  0.8, 1.1 | 0.7  1.2  0.6  0.9  1.6  1.4 | 0.6, 0.8  1.1, 1.4  0.5, 0.6  0.8, 1.0  1.5, 1.7  1.3, 1.6 | 1.0  2.0  0.6  1.1  1.9  0.9 | 0.8, 1.2  1.5, 2.4  0.5, 0.7  0.9, 1.3  1.7, 2.0  0.7, 1.0 | 0.7  1.2  0.5  0.9  1.5  1.3 | 0.6, 0.8  1.0, 1.4  0.4, 0.6  0.8, 1.0  1.4, 1.6  1.2, 1.5 | 0.9  1.5  0.6  1.1  1.6  0.9 | 0.8, 1.1  1.3, 1.8  0.5, 0.7  0.9, 1.2  1.4, 1.8  0.7, 1.1 |
| Dinner (serve/s) | | | | | | | | | | | | |
| Fruit  Vegetables  Dairy  Proteins  Grains  Discret. | 0.7  1.8  0.6  1.4  2.1  2.5 | 0.5, 0.8  1.7, 1.9  0.6, 0.7  1.3, 1.5  2.0, 2.2  2.3, 2.7 | 0.6  2.8  0.6  1.6  2.1  1.5 | 0.5, 0.8  2.6, 3.0  0.5, 0.7  1.5, 1.6  1.9, 2.3  1.3, 1.7 | 0.6  2.1  0.6  1.4  1.8  2.2 | 0.5, 0.8  1.8, 2.3  0.5, 0.7  1.3, 1.5  1.7, 2.0  2.0, 2.4 | 0.7  3.1  0.6  1.8  1.9  1.5 | 0.6, 0.8  2.8, 3.4  0.5, 0.7  1.6, 1.9  1.7, 2.1  1.3, 1.8 | 0.6  2.0  0.5  1.3  1.6  1.8 | 0.5, 0.7  1.7, 2.3  0.4, 0.6  1.2, 1.4  1.4, 1.7  1.5, 2.0 | 1.0  2.8  0.6  1.6  1.3  1.5 | 0.8, 1.2  2.5, 3.2  0.5, 0.7  1.4, 1.7  1.1, 1.5  1.3, 1.8 |
| Snack (serve/s) | | | | | | | | | | | | |
| Fruit  Vegetables  Dairy  Proteins  Grains  Discret. | 1.1  0.9  0.7  0.6  1.2  2.9 | 1.0, 1.2 0.7, 1.1  0.7, 0.8  0.5, 0.7  1.1, 1.3  2.7, 3.1 | 1.5  1.0  0.9  0.7  1.0  1.9 | 1.4, 1.7  0.8, 1.3  0.8, 1.0  0.6, 0.9  0.9, 1.2  1.7, 2.0 | 1.2  0.8  0.7  0.5  1.0  2.5 | 1.0, 1.4  0.6, 1.0  0.6, 0.8  0.4, 0.6  0.9, 1.2  2.2, 2.7 | 1.5  1.0  0.8  0.9  0.9  1.4 | 1.3, 1.7  0.7, 1.2  0.7, 0.9  0.7, 1.1  0.8, 1.1  1.2, 1.6 | 0.9  0.7  0.6  0.2  0.8  2.0 | 0.7, 1.0  0.4, 1.0  0.5, 0.7  0.2, 0.3  0.7, 0.9  1.7, 2.3 | 1.3  1.1  0.7  0.5  1.1  1.4 | 1.1, 1.6  -0.1, 2.3  0.6, 0.8  0.3, 0.7  0.7, 1.4  1.1, 1.7 |
| Total (serve/s) | | | | | | | | | | | | |
| Fruit  Vegetables  Dairy  Proteins  Grains  Discret. | 1.5  2.7  1.6  2.1  4.7  5.3 | 1.4, 1.6  2.5, 2.8  1.5, 1.7  2.0, 2.2  4.5, 4.8  5.0, 5.5 | 2.2  4.2  1.9  2.6  5.0  3.0 | 2.0, 2.3  4.0, 4.5  1.8, 2.0  2.4, 2.8  4.7, 5.3  2.8, 3.2 | 1.6  2.8  1.5  2.1  4.4  4.6 | 1.4, 1.8  2.6, 3.0  1.4, 1.6  2.0, 2.2  4.1, 4.6  4.3, 4.9 | 2.4  4.7  1.8  2.8  4.7  2.5 | 2.2, 2.6  4.2, 5.2  1.7, 1.9  2.6, 3.1  4.3, 5.0  2.2, 2.7 | 1.4  2.5  1.4  1.8  4.1  4.1 | 1.3, 1.6  2.2, 2.8  1.3, 1.5  1.7, 1.9  3.8, 4.3  3.8, 4.5 | 2.3  3.8  1.6  2.5  4.2  2.7 | 2.0, 2.7  3.4, 4.2  1.4, 1.8  2.3, 2.7  3.8, 4.5  2.4, 2.9 |

^1^ Weighted mean (in serve/s) of food consumption when consumption of food groups occurred. Non-consumers (for each food group and eating occasion) were not included in analysis.

^2^ Higher diet quality (DQ) – the top tertile of dietary guidelines index score (0-130) which assessed adherence to the Australian Dietary Guidelines. Lower diet quality (DQ) – bottom two tertiles of the score.

All results were weighted to be nationally representative of Australian population.
