# Supplementary material for: Achieving high diet quality at eating occasions: findings from a nationally representative study of Australian adults
Source: Br J Nutr. 2023 Oct 19;131(5):868–79. doi: 10.1017/S0007114523002325 (PMC10864991; doi:10.1017/S0007114523002325)
Supplement: Tran et al. supplementary material 4 — Tran et al. supplementary material [file S0007114523002325sup004.docx]

**Additional file 4. Weighted means in serves of food group consumption at eating occasions between Australian men with low level of adherence and high level of adherence to dietary guidelines^2^, stratified by age group. (n = 4245)**

|  | 19-50 years | | | | 51-70 years | | | | 71 years and over | | | |
| --- | --- | --- | --- | --- | --- | --- | --- | --- | --- | --- | --- | --- |
| n (person) | 2380 | | | | 1334 | | | | 531 | | | |
|  | **Low**er  DQ | | **High**er  **DQ** | | **Low**er  DQ | | **High**er  **DQ** | | **Low**er  DQ | | **High**er  **DQ** | |
| n (person) | 1673 | | 707 | | 973 | | 361 | | 372 | | 159 | |
|  | **Mean** | **95%**  **CI** | **Mean** | **95%**  **CI** | **Mean** | **95%**  **CI** | **Mean** | **95%**  **CI** | **Mean** | **95%**  **CI** | **Mean** | **95%**  **CI** |
| Breakfast (serve/s) | | | | | | | | | | | | |
| Fruit  Vegetables  Dairy  Proteins  Grains  Discret. | 1.2  1.3  0.8  1.0  2.1  1.2 | 1.0, 1.4  1.0, 1.7  0.8, 0.9  0.8, 1.1  2.0, 2.2  1.1, 1.3 | 1.5  1.3  1.0  0.9  2.4  0.9 | 1.3, 1.7  1.0, 1.7  0.9, 1.1  0.7, 1.0  2.3, 2.6  0.8, 1.1 | 0.9  1.1  0.6  0.9  2.1  0.9 | 0.8, 1.0  0.9, 1.3  0.5, 0.6  0.8, 1.0  1.9, 2.3  0.8, 1.1 | 1.3  1.4  0.9  0.9  2.5  0.6 | 1.0, 1.5  0.8, 2.0  0.8, 1.0  0.7, 1.1  2.3, 2.7  0.5, 0.7 | 0.9  1.1  0.5  0.7  2.0  0.8 | 0.8, 1.1  0.6, 1.5  0.5, 0.6  0.5, 0.9  1.9, 2.1  0.6, 0.9 | 1.5  1.3  0.7  0.9  2.5  0.6 | 1.0, 2.0  0.8, 1.8  0.6, 0.8  0.6, 1.3  2.2, 2.8  0.4, 0.7 |
| Lunch (serve/s) | | | | | | | | | | | | |
| Fruit  Vegetables  Dairy  Proteins  Grains  Discret. | 0.8  1.3  0.8  1.3  2.6  2.4 | 0.7, 1.0  1.2, 1.4  0.7, 0.8  1.2, 1.4  2.5, 2.8  2.1, 2.7 | 1.2  1.9  0.9  1.5  2.6  1.4 | 1.0, 1.4  1.5, 2.2  0.7, 1.0  1.3, 1.6  2.4, 2.7  1.1, 1.6 | 0.8  1.3  0.7  1.2  2.2  1.7 | 0.7, 1.0  1.1, 1.5  0.6, 0.8  1.1, 1.4  2.1, 2.4  1.5, 1.9 | 1.2  1.8  0.8  1.3  2.2  1.1 | 1.0, 1.4  1.5, 2.2  0.6, 0.9  1.1, 1.4  2.0, 2.4  0.8, 1.3 | 0.9  1.6  0.5  1.1  2.0  1.7 | 0.7, 1.0  1.3, 1.9  0.4, 0.6  1.0, 1.2  1.8, 2.1  1.5, 2.0 | 1.5  2.1  0.7  1.0  2.1  1.2 | 1.0, 2.0  1.4, 2.7  0.5, 1.0  0.8, 1.2  1.9, 2.4  0.7, 1.7 |
| Dinner (serve/s) | | | | | | | | | | | | |
| Fruit  Vegetables  Dairy  Proteins  Grains  Discret. | 0.5  2.3  0.8  2.0  2.7  3.1 | 0.4, 0.5  2.1, 2.4  0.7, 0.8  1.9, 2.1  2.6, 2.9  2.8, 3.3 | 0.8  3.2  0.8  2.1  2.9  1.8 | 0.7, 1.0  2.9, 3.5  0.7, 0.9  1.9, 2.2  2.6, 3.2  1.6, 2.0 | 0.7  2.4  0.8  1.8  2.4  3.0 | 0.5, 0.8  2.2, 2.6  0.7, 0.9  1.7, 2.0  2.1, 2.6  2.8, 3.2 | 1.1  3.6  0.7  2.0  2.4  2.1 | 0.9, 1.2  3.3, 3.9  0.5, 0.8  1.8, 2.3  2.2, 2.7  1.6, 2.5 | 0.8  2.2  0.6  1.5  1.9  2.6 | 0.6, 1.1  1.9, 2.5  0.5, 0.6  1.3, 1.7  1.7, 2.1  2.2, 3.1 | 1.2  3.4  0.6  1.7  1.5  1.8 | 0.9, 1.5  2.9, 3.8  0.5, 0.7  1.5, 1.8  1.2, 1.9  1.4, 2.2 |
| Snack (serve/s) | | | | | | | | | | | | |
| Fruit  Vegetables  Dairy  Proteins  Grains  Discret. | 1.5  1.2  1.0  0.8  1.8  4.2 | 1.3, 1.6  1.0, 1.4  0.9, 1.1  0.7, 0.9  1.6, 2.0  3.9, 4.4 | 1.8  1.4  1.1  1.3  1.7  2.2 | 1.6, 2.0  1.1, 1.8  1.0, 1.3  1.1, 1.5  1.5, 2.0  2.0, 2.5 | 1.2  1.2  0.8  0.8  1.3  3.4 | 1.0, 1.4  0.5, 1.9  0.7, 0.9  0.6, 0.9  1.1, 1.5  3.1, 3.7 | 1.9  0.9  0.8  1.2  1.5  2.1 | 1.6, 2.3  0.6, 1.3  0.7, 0.9  0.9, 1.5  1.2, 1.8  1.8, 2.4 | 1.1  1.5  0.6  0.3  1.2  2.4 | 0.8, 1.3  0.6, 2.4  0.4, 0.7  0.2, 0.4  1.0, 1.4  2.1, 2.7 | 1.5  1.1  0.7  0.7  0.7  1.9 | 1.2, 1.8  0.3, 1.8  0.5, 1.0  0.4, 0.9  0.6, 0.9  1.3, 2.4 |
| Total (serve/s) | | | | | | | | | | | | |
| Fruit  Vegetables  Dairy  Proteins  Grains  Discret. | 1.8  3.3  1.9  3.2  6.4  7.4 | 1.6,1.9  3.1, 3.4  1.8, 2.1  3.0, 3.4  6.2, 6.7  7.4, 8.0 | 2.8  4.8  2.4  3.9  7.2  3.9 | 2.6, 3.0  4.3, 5.2  2.2, 2.5  3.7, 4.1  6.8, 7.6  3.6, 4.3 | 1.7  3.1  1.6  3.0  5.6  6.4 | 1.5, 1.8  2.9, 3.4  1.5, 1.8  2.8, 3.2  5.3, 6.0  6.1, 6.7 | 3.0  5.0  2.0  3.5  6.5  3.6 | 2.7, 3.3  4.6, 5.4  1.8, 2.1  3.2, 3.7  6.0, 6.9  3.2, 4.0 | 1.8  3.1  1.4  2.4  5.3  5.5 | 1.6, 2.1  2.8, 3.5  1.3, 1.5  2.2, 2.6  5.0, 5.7  4.9, 6.0 | 3.0  4.7  1.8  2.7  5.6  3.4 | 2.5, 3.4  4.1, 5.3  1.6, 2.1  2.4, 3.0  5.2, 6.0  2.8, 4.1 |

^1^ Weighted mean (in serve/s) of food consumption when consumption of food groups occurred. Non-consumers (for each food group and eating occasion) were not included in analysis.

^2^ Higher diet quality (DQ) – the top tertile of dietary guidelines index score (0-130) which assessed adherence to the Australian Dietary Guidelines. Lower diet quality (DQ) – bottom two tertiles of the score.

All results were weighted to be nationally representative of Australian population.
