# Supplementary material for: Achieving high diet quality at eating occasions: findings from a nationally representative study of Australian adults
Source: Br J Nutr. 2023 Oct 19;131(5):868–79. doi: 10.1017/S0007114523002325 (PMC10864991; doi:10.1017/S0007114523002325)
Supplement: Tran et al. supplementary material 5 — Tran et al. supplementary material [file S0007114523002325sup005.docx]

**Additional file 5 – Means^1^ in serves of food group consumption at eating occasions between Australian women with lower and higher level of diet quality^2^, stratified by education level, adjusted for age. (n = 4809)**

|  | Low level of Education | | | | Medium level of education | | | | High level of Education | | | |
| --- | --- | --- | --- | --- | --- | --- | --- | --- | --- | --- | --- | --- |
| n (person) | 1534 | | | | 1992 | | | | 1283 | | | |
|  | **Low**er DQ | | **High**er DQ | | **Low**er DQ | | **High**er DQ | | **Low**er DQ | | **High**er DQ | |
| n (person) | 1053 | | 481 | | 1279 | | 713 | | 687 | | 596 | |
|  | **Mean** | **95 % CI** | **Mean** | **95 % CI** | **Mean** | **95 % CI** | **Mean** | **95 % CI** | **Mean** | **95 % CI** | **Mean** | **95 % CI** |
| Breakfast (serve/s) | | | | | | | | | | | | |
| Fruit | 0.3 | 0.3, 0.4 | 0.5 | 0.4, 0.5 | 0.4 | 0.3, 0.4 | 0.5 | 0.5, 0.6 | 0.3 | 0.3, 0.4 | 0.5 | 0.4, 0.5 |
| Vegetables | 0.1 | 0.0, 0.1 | 0.2 | 0.1, 0.2 | 0.1 | 0.1, 0.2 | 0.2 | 0.1, 0.3 | 0.1 | 0.1, 0.2 | 0.2 | 0.1, 0.3 |
| Dairy | 0.4 | 0.4, 0.5 | 0.6 | 0.5, 0.6 | 0.4 | 0.4, 0.5 | 0.6 | 0.5, 0.6 | 0.4 | 0.4, 0.5 | 0.6 | 0.5, 0.6 |
| Proteins | 0.1 | 0.1, 0.2 | 0.2 | 0.1, 0.2 | 0.2 | 0.1, 0.2 | 0.2 | 0.2, 0.2 | 0.2 | 0.1, 0.2 | 0.2 | 0.2, 0.2 |
| Grains | 1.4 | 1.3, 1.4 | 1.6 | 1.5, 1.7 | 1.3 | 1.2, 1.4 | 1.5 | 1.4, 1.6 | 1.3 | 1.2, 1.4 | 1.5 | 1.4, 1.6 |
| Discret. | 0.4 | 0.3, 0.4 | 0.2 | 0.2, 0.3 | 0.4 | 0.4, 0.5 | 0.3 | 0.2, 0.3 | 0.4 | 0.4, 0.5 | 0.3 | 0.2, 0.3 |
| Lunch (serve/s) | | | | | | | | | | | | |
| Fruit | 0.2 | 0.2, 0.3 | 0.4 | 0.3, 0.4 | 0.2 | 0.2, 0.2 | 0.3 | 0.3, 0.4 | 0.3 | 0.2, 0.3 | 0.4 | 0.3, 0.5 |
| Vegetables | 0.7 | 0.6, 0.8 | 1.2 | 1.0, 1.4 | 0.8 | 0.7, 0.9 | 1.3 | 1.1, 1.5 | 1.0 | 0.8, 1.1 | 1.4 | 1.3, 1.6 |
| Dairy | 0.3 | 0.2, 0.3 | 0.3 | 0.3, 0.3 | 0.3 | 0.3, 0.4 | 0.3 | 0.3, 0.4 | 0.3 | 0.3, 0.4 | 0.3 | 0.3, 0.4 |
| Proteins | 0.6 | 0.6, 0.7 | 0.7 | 0.6, 0.8 | 0.7 | 0.6, 0.7 | 0.8 | 0.7, 0.8 | 0.6 | 0.6, 0.7 | 0.7 | 0.6, 0.8 |
| Grains | 1.4 | 1.3, 1.5 | 1.4 | 1.3, 1.5 | 1.4 | 1.3, 1.5 | 1.5 | 1.4, 1.6 | 1.6 | 1.4, 1.7 | 1.6 | 1.5, 1.7 |
| Discret. | 0.1 | 0.9, 1.1 | 0.5 | 0.3, 0.6 | 1.1 | 1.0, 1.2 | 0.6 | 0.5, 0.7 | 0.9 | 0.8, 1.0 | 0.4 | 0.3, 0.5 |
| Dinner (serve/s) | | | | | | | | | | | | |
| Fruit | 0.2 | 0.1, 0.2 | 0.2 | 0.2, 0.3 | 0.2 | 0.2, 0.3 | 0.3 | 0.2, 0.3 | 0.2 | 0.2, 0.3 | 0.3 | 0.2, 0.3 |
| Vegetables | 1.7 | 1.5, 1.9 | 2.7 | 2.5, 2.9 | 1.6 | 1.5, 1.8 | 2.7 | 2.5, 2.8 | 1.7 | 1.6, 1.8 | 2.7 | 2.5, 3.0 |
| Dairy | 0.3 | 0.3, 0.4 | 0.3 | 0.2, 0.3 | 0.3 | 0.3, 0.4 | 0.3 | 0.2, 0.3 | 0.3 | 0.3, 0.4 | 0.3 | 0.2, 0.3 |
| Proteins | 1.2 | 1.1, 1.3 | 1.5 | 1.4, 1.6 | 1.2 | 1.1, 1.3 | 1.5 | 1.4, 1.6 | 1.1 | 1.0, 1.2 | 1.4 | 1.3, 1.5 |
| Grains | 1.3 | 1.2, 1.4 | 1.2 | 1.0, 1.3 | 1.5 | 1.4, 1.5 | 1.4 | 1.3, 1.5 | 1.6 | 1.4, 1.7 | 1.4 | 1.3, 1.6 |
| Discret. | 1.5 | 1.3, 1.6 | 0.7 | 0.5, 0.6 | 1.6 | 1.4, 1.7 | 0.8 | 0.7, 0.9 | 1.7 | 1.5, 1.8 | 0.9 | 0.7, 1.0 |
| Snack (serve/s) | | | | | | | | | | | | |
| Fruit | 0.5 | 0.4, 0.6 | 1.0 | 0.9, 1.1 | 0.6 | 0.5, 0.6 | 1.1 | 1.0, 1.2 | 0.7 | 0.6, 0.7 | 1.2 | 1.1, 1.3 |
| Vegetables | 0.1 | 0.1, 0.2 | 0.2 | 0.1, 0.2 | 0.2 | 0.1, 0.2 | 0.2 | 0.2, 0.3 | 0.2 | 0.1, 0.3 | 0.2 | 0.2, 0.3 |
| Dairy | 0.5 | 0.4, 0.5 | 0.6 | 0.5, 0.7 | 0.5 | 0.5, 0.6 | 0.7 | 0.6, 0.7 | 0.6 | 0.5, 0.6 | 0.7 | 0.6, 0.7 |
| Proteins | 0.1 | 0.1, 0.2 | 0.3 | 0.2, 0.3 | 0.2 | 0.2, 0.2 | 0.3 | 0.3, 0.4 | 0.2 | 0.2, 0.3 | 0.3 | 0.3, 0.4 |
| Grains | 0.6 | 0.5, 0.6 | 0.5 | 0.4, 0.5 | 0.7 | 0.6, 0.7 | 0.6 | 0.5, 0.6 | 0.7 | 0.5, 0.8 | 0.6 | 0.5, 0.7 |
| Discret. | 2.0 | 1.8, 2.2 | 1.0 | 0.8, 1.2 | 2.3 | 2.1, 2.5 | 1.3 | 1.2, 1.5 | 2.3 | 2.1, 2.4 | 1.3 | 1.1, 1.5 |

^1^Marginal means were adjusted for age group, using survey design based generalised linear regression. Non-consumers (for each food group and eating occasion) were not included in analysis.

^2^Higher diet quality (DQ) – the top tertile of dietary guidelines index score (0-130) which assessed adherence to the Australian Dietary Guidelines. Lower diet quality – bottom two tertiles of the score.
