# Supplementary material for: Achieving high diet quality at eating occasions: findings from a nationally representative study of Australian adults
Source: Br J Nutr. 2023 Oct 19;131(5):868–79. doi: 10.1017/S0007114523002325 (PMC10864991; doi:10.1017/S0007114523002325)
Supplement: Tran et al. supplementary material 6 — Tran et al. supplementary material [file S0007114523002325sup006.docx]

**Additional file 6 – Means^1^ in serves of food group consumption at eating occasions between Australian men with low level of adherence and high level of adherence to dietary guideline3, stratified by education level, adjusted for age. (n = 4245)**

|  | Low level of Education | | | | Medium level of education | | | | High level of Education | | | |
| --- | --- | --- | --- | --- | --- | --- | --- | --- | --- | --- | --- | --- |
| n (person) | 1039 | | | | 2198 | | | | 1008 | | | |
|  | **Low**er DQ | | **High**er DQ | | **Low**er DQ | | **High**er DQ | | **Low**er DQ | | **High**er DQ | |
| n (person) | 815 | | 224 | | 1569 | | 629 | | 634 | | 374 | |
|  | **Mean** | **95 % CI** | **Mean** | **95 % CI** | **Mean** | **95 % CI** | **Mean** | **95 % CI** | **Mean** | **95 % CI** | **Mean** | **95 % CI** |
| Breakfast (serve/s) | | | | | | | | | | | | |
| Fruit | 0.3 | 0.2, 0.4 | 0.7 | 0.5, 0.8 | 0.4 | 0.4, 0.5 | 0.8 | 0.7, 0.9 | 0.6 | 0.4, 0.7 | 0.9 | 0.8, 1.0 |
| Vegetables | 0.1 | 0.1, 0.2 | 0.2 | 0.1, 0.3 | 0.2 | 0.1, 0.2 | 0.2 | 0.2, 0.3 | 0.2 | 0.1, 0.3 | 0.3 | 0.2, 0.4 |
| Dairy | 0.5 | 0.4, 0.5 | 0.7 | 0.6, 0.7 | 0.6 | 0.5, 0.6 | 0.8 | 0.7, 0.8 | 0.6 | 0.5, 0.6 | 0.8 | 0.7, 0.9 |
| Proteins | 0.3 | 0.2, 0.4 | 0.3 | 0.2, 0.4 | 0.3 | 0.3, 0.4 | 0.3 | 0.3, 0.4 | 0.4 | 0.3, 0.4 | 0.4 | 0.3, 0.4 |
| Grains | 1.9 | 1.7, 2.1 | 2.3 | 2.1, 2.5 | 1.8 | 1.7, 1.9 | 2.2 | 2.1, 2.4 | 1.8 | 1.7, 2.0 | 2.3 | 2.1, 2.5 |
| Discret. | 0.8 | 0.7, 0.9 | 0.6 | 0.4, 0.7 | 0.7 | 0.6, 0.8 | 0.4 | 0.4, 0.5 | 0.6 | 0.5, 0.7 | 0.4 | 0.3, 0.5 |
| Lunch (serve/s) | | | | | | | | | | | | |
| Fruit | 0.3 | 0.3, 0.4 | 0.5 | 0.4, 0.6 | 0.3 | 0.2, 0.3 | 0.5 | 0.4, 0.5 | 0.2 | 0.2, 0.3 | 0.4 | 0.3, 0.5 |
| Vegetables | 0.9 | 0.8, 1.0 | 1.3 | 1.1, 1.5 | 0.9 | 0.7, 1.0 | 1.3 | 1.1, 1.5 | 1.2 | 1.0, 1.3 | 1.6 | 1.3, 1.9 |
| Dairy | 0.3 | 0.3, 0.4 | 0.4 | 0.3, 0.4 | 0.3 | 0.3, 0.4 | 0.4 | 0.3, 0.5 | 0.3 | 0.3, 0.4 | 0.4 | 0.3, 0.5 |
| Proteins | 0.9 | 0.8, 1.0 | 1.0 | 0.9, 1.1 | 1.0 | 0.9, 1.1 | 1.1 | 1.0, 1.2 | 1.1 | 1.0, 1.2 | 1.2 | 1.0, 1.3 |
| Grains | 2.0 | 1.8, 2.1 | 1.9 | 1.7, 2.1 | 2.1 | 1.9, 2.2 | 2.0 | 1.9, 2.1 | 2.3 | 2.1, 2.5 | 2.3 | 2.0, 2.5 |
| Discret. | 1.5 | 1.3, 1.7 | 0.7 | 0.5, 1.0 | 1.4 | 1.3, 1.6 | 0.7 | 0.5, 0.8 | 1.4 | 1.2, 1.6 | 0.6 | 0.5, 0.8 |
| Dinner (serve/s) | | | | | | | | | | | | |
| Fruit | 0.2 | 0.2, 0.3 | 0.4 | 0.3, 0.4 | 0.2 | 0.1, 0.2 | 0.3 | 0.3, 0.4 | 0.2 | 0.1, 0.2 | 0.3 | 0.3, 0.4 |
| Vegetables | 2.0 | 1.8, 2.2 | 3.0 | 2.8, 3.3 | 2.0 | 1.8, 2.1 | 3.0 | 2.8, 3.2 | 2.1 | 1.8, 2.4 | 3.2 | 2.8, 3.5 |
| Dairy | 0.3 | 0.3, 0.4 | 0.3 | 0.3, 0.4 | 0.4 | 0.3, 0.4 | 0.4 | 0.3, 0.4 | 0.3 | 0.3, 0.4 | 0.4 | 0.3, 0.4 |
| Proteins | 1.8 | 1.6, 1.9 | 1.9 | 1.7, 2.1 | 1.7 | 1.6, 1.8 | 1.8 | 1.7, 2.0 | 1.6 | 1.4, 1.7 | 1.7 | 1.6, 1.9 |
| Grains | 1.7 | 1.5, 1.8 | 1.6 | 1.3, 1.8 | 2.0 | 1.8, 2.1 | 1.9 | 1.7, 2.0 | 2.3 | 2.1, 2.5 | 2.2 | 2.0, 2.5 |
| Discret. | 2.0 | 1.8, 2.2 | 0.8 | 0.6, 1.1 | 2.2 | 2.0, 2.4 | 1.1 | 0.9, 1.2 | 2.0 | 1.8, 2.2 | 0.8 | 0.6, 1.0 |
| Snack (serve/s) | | | | | | | | | | | | |
| Fruit | 0.6 | 0.5, 0.7 | 1.2 | 1.0, 1.3 | 0.6 | 0.5, 0.7 | 1.2 | 1.1, 1.3 | 0.6 | 0.5, 0.7 | 1.2 | 1.0, 1.4 |
| Vegetables | 0.2 | 0.2, 0.3 | 0.3 | 0.2, 0.4 | 0.3 | 0.2, 0.4 | 0.3 | 0.3, 0.4 | 0.2 | 0.1, 0.3 | 0.2 | 0.1, 0.3 |
| Dairy | 0.6 | 0.5, 0.7 | 0.6 | 0.5, 0.8 | 0.7 | 0.6, 0.7 | 0.7 | 0.7, 0.8 | 0.6 | 0.5, 0.7 | 0.7 | 0.6, 0.8 |
| Proteins | 0.2 | 0.2, 0.3 | 0.5 | 0.4, 0.6 | 0.3 | 0.2, 0.3 | 0.5 | 0.4, 0.6 | 0.3 | 0.2, 0.4 | 0.5 | 0.4, 0.6 |
| Grains | 0.9 | 0.7, 1.0 | 0.9 | 0.7, 0.9 | 1.0 | 0.9, 1.1 | 0.9 | 0.8, 1.1 | 1.7 | 1.6, 0.9 | 0.7 | 0.5, 0.9 |
| Discret. | 3.3 | 2.9, 3.7 | 1.8 | 1.4, 2.1 | 3.4 | 3.1, 3.6 | 1.8 | 1.6, 2.1 | 2.9 | 2.6, 3.1 | 1.3 | 1.1, 1.5 |

^1^Marginal means were adjusted for age group, using survey design based generalised linear regression. Non-consumers (for each food group and eating occasion) were not included in analysis.

^2^Higher diet quality (DQ) – the top tertile of dietary guidelines index score (0-130) which assessed adherence to the Australian Dietary Guidelines. Lower diet quality – bottom two tertiles of the score.
